# Supplementary material for: The key role of Au-substrate interactions in catalytic gold subnanoclusters
Source: Nat Commun. 2017 Nov 21;8:1657. doi: 10.1038/s41467-017-01675-1 (PMC5698477; doi:10.1038/s41467-017-01675-1)
Supplement: Supplementary file 1 — Supplementary Information [file 41467_2017_1675_MOESM1_ESM.pdf]

Supplementary Figures

a

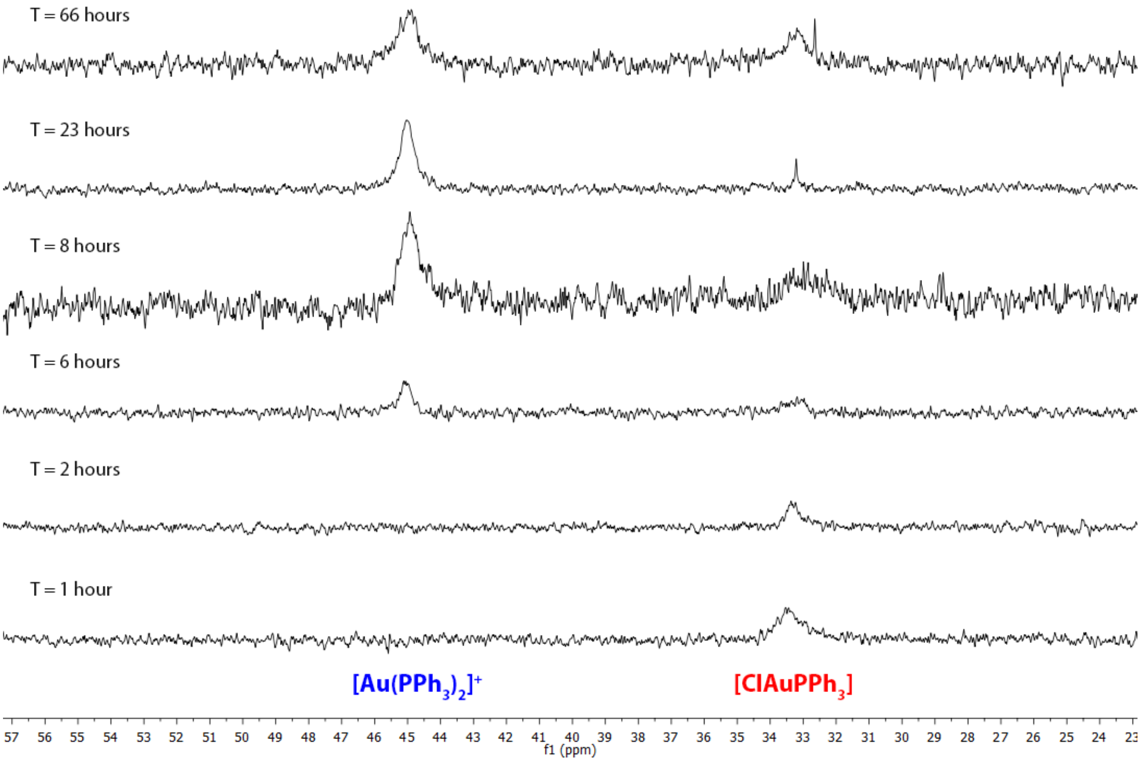

b

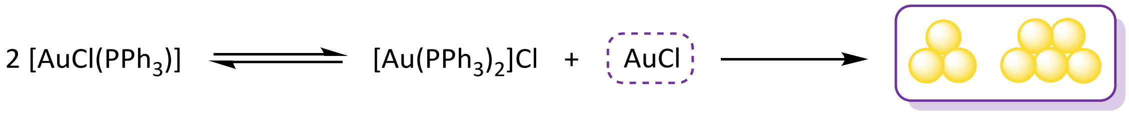

Supplementary Figure 1. a, $^{31}P\{^{1}H\}$ NMR monitoring of the hydration reaction of phenylacetylene (**4**) using 4 mol% of $[AuCl(PPh_3)]$ as a precatalyst showing the slow formation of concomitant [Au(PPh3)2]Cl and AuCl. **b,** Formation of gold subnanoclusters from $[AuCl(PPh_3)]$.

a

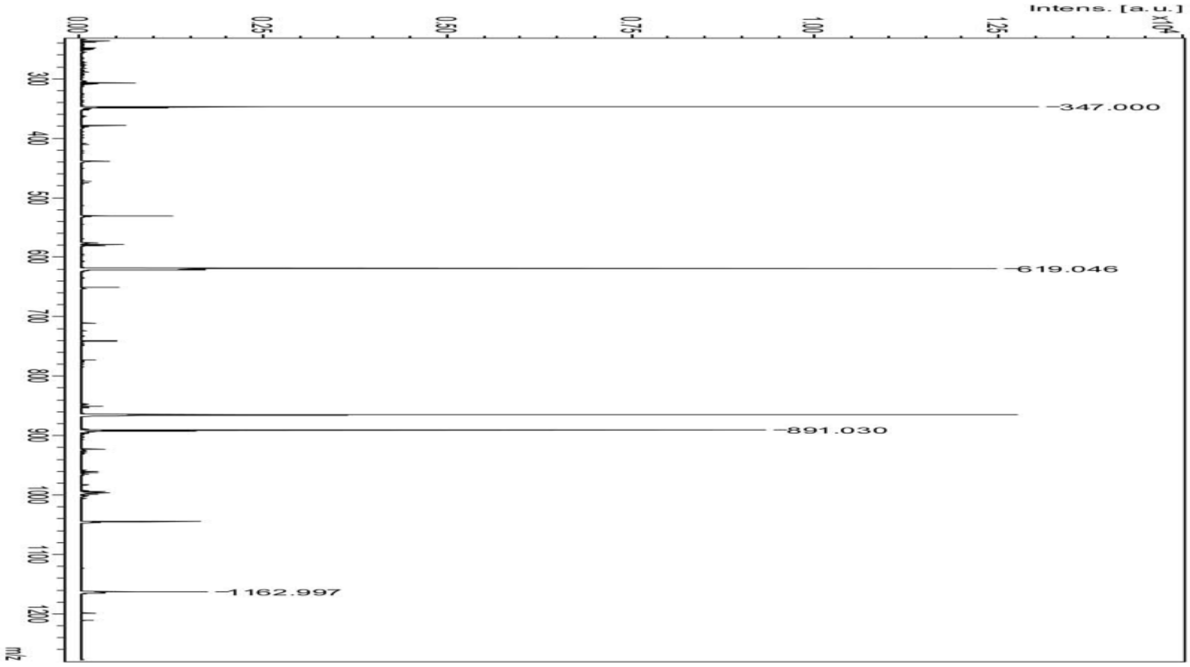

b

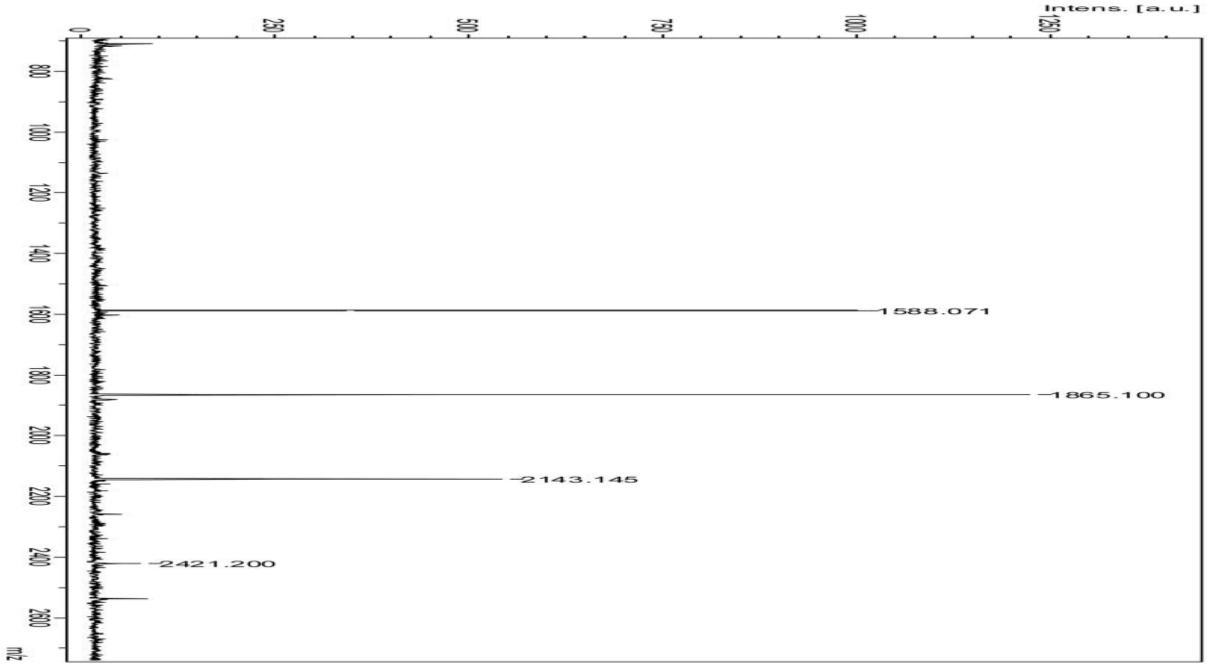

Supplementary Figure 2. MALDI-TOF spectra showing the addition of **a**, thiols or **b**, amines (bottom) to 1-hexyne using using 2% mol of AuCl (**I**) as a precatalyst. In the addition of propanethiol we observe in the MALDI – spectrum (up) the peaks corresponding to m/z = 347 $[Au(SR)_2]^-$; m/z = 619 $[Au_2(SR)_3]^-$; m/z = 891 $[Au_3(SR)_4]^-$; m/z = 1163 $[Au_4(SR)_5]^-$. In the addition of propylamine we observe in the MALDI + spectrum (bottom) the peaks corresponding to m/z = 1588 $[Au_6(\text{1-hexyne-H})_5]^+$; m/z = 1865 $[Au_7(\text{1-hexyne-H})_6]^+$; m/z = 2143 $[Au_8(\text{1-hexyne-H})_7]^+$; m/z = 2421 $[Au_9(\text{1-hexyne-H})_8]^+$.

a

b

c

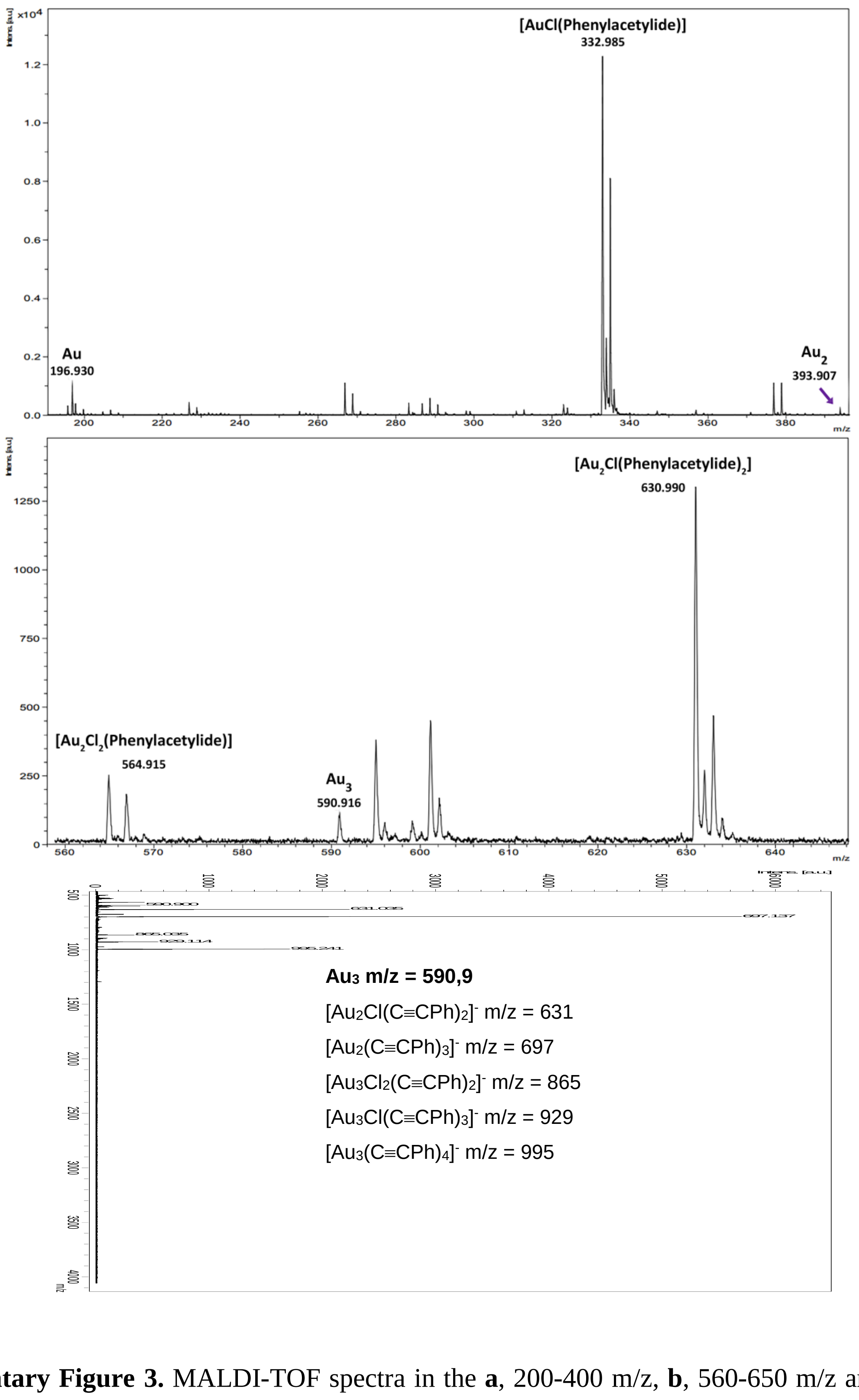

Supplementary Figure 3. MALDI-TOF spectra in the **a**, 200-400 m/z, **b**, 560-650 m/z and **c**, 500-4000 m/z ranges, showing the formation of small size clusters (mainly Au-Au_3) in the hydration of phenylacetylene (**4**), using 2% mol of AuCl (**I**) as a precatalyst, at the beginning of the reaction (55 min).

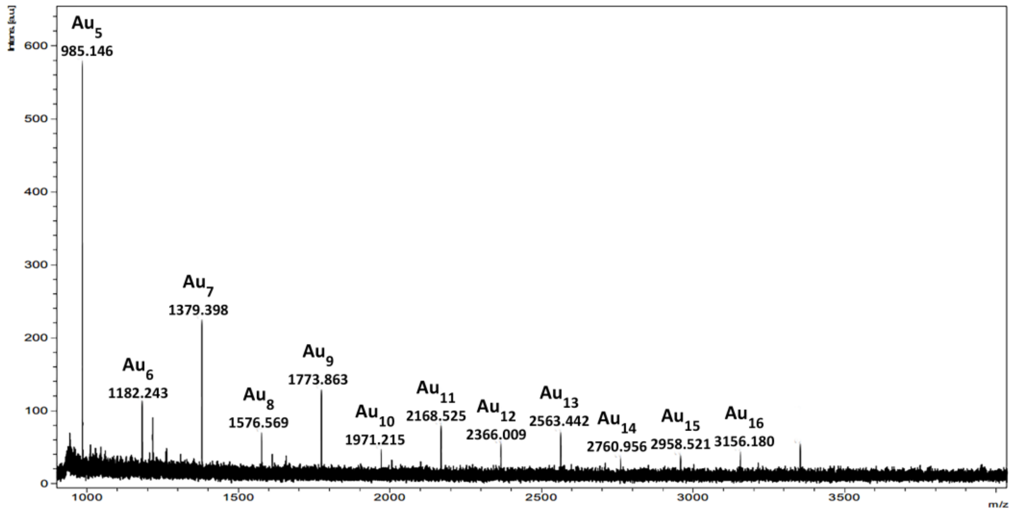

Supplementary Figure 4. MALDI-TOF spectrum showing the formation of larger size clusters (mainly Au_5-Au_{17}) in the hydration of phenylacetylene (**4**), using 2% mol of AuCl (**I**) as a precatalyst, at very long reaction times (262 h).

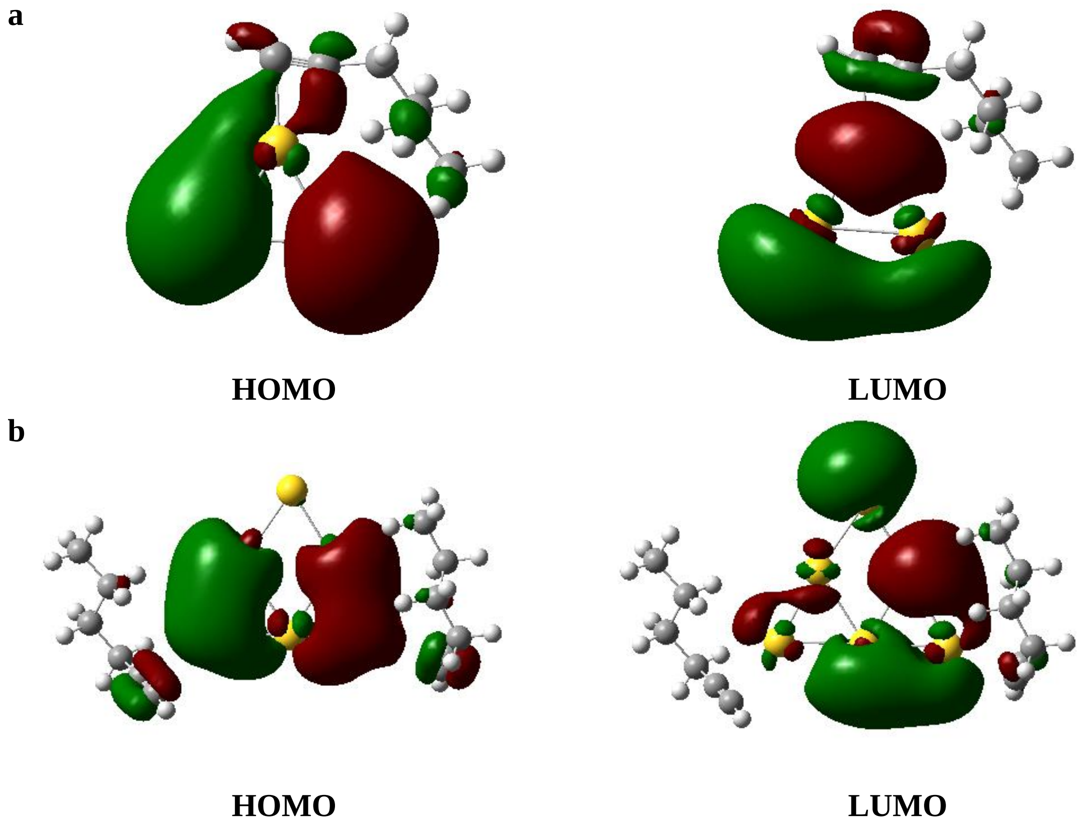

Supplementary Figure 5: HOMO and LUMO orbitals of model systems **a**, [Au_3(1-hexyne)] and **b**, [Au_6(1-hexyne)$_2$] computed through DFT.

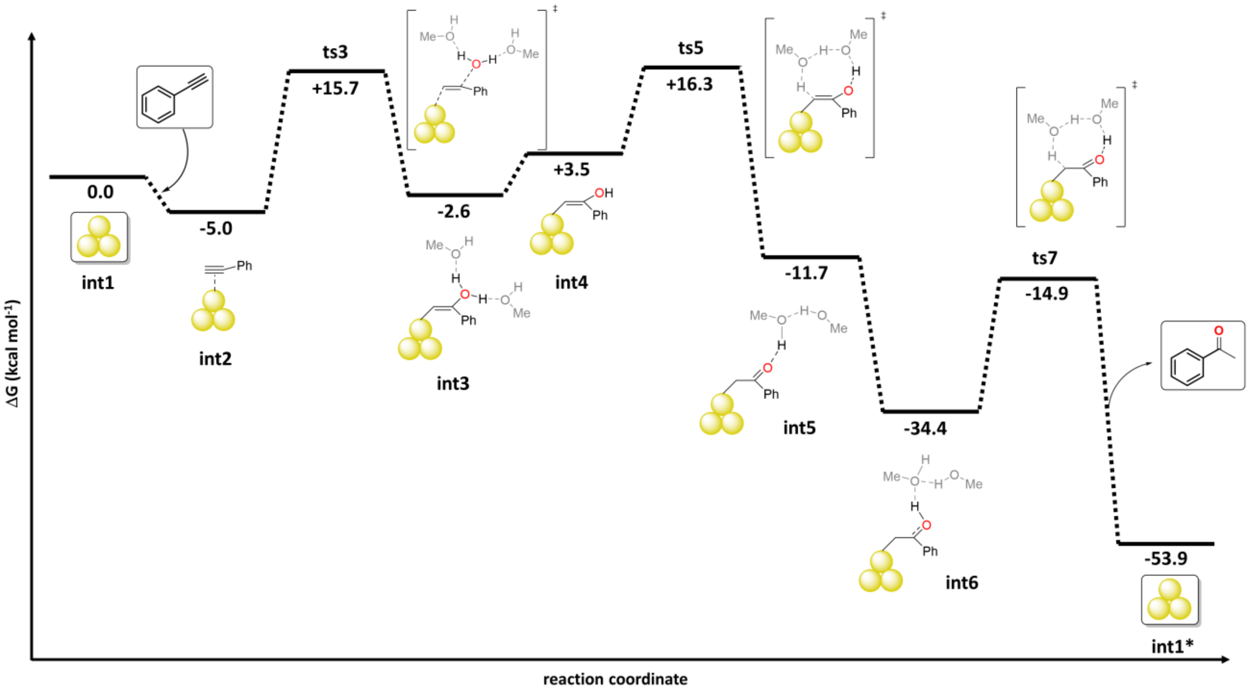

Supplementary Figure 6. Minimum-energy reaction pathway for the Au_3-catalyzed hydration of phenylacetylene calculated with M06-2X/SDD. Relative Gibbs free energies (ΔG) are in kcal mol^{-1}.

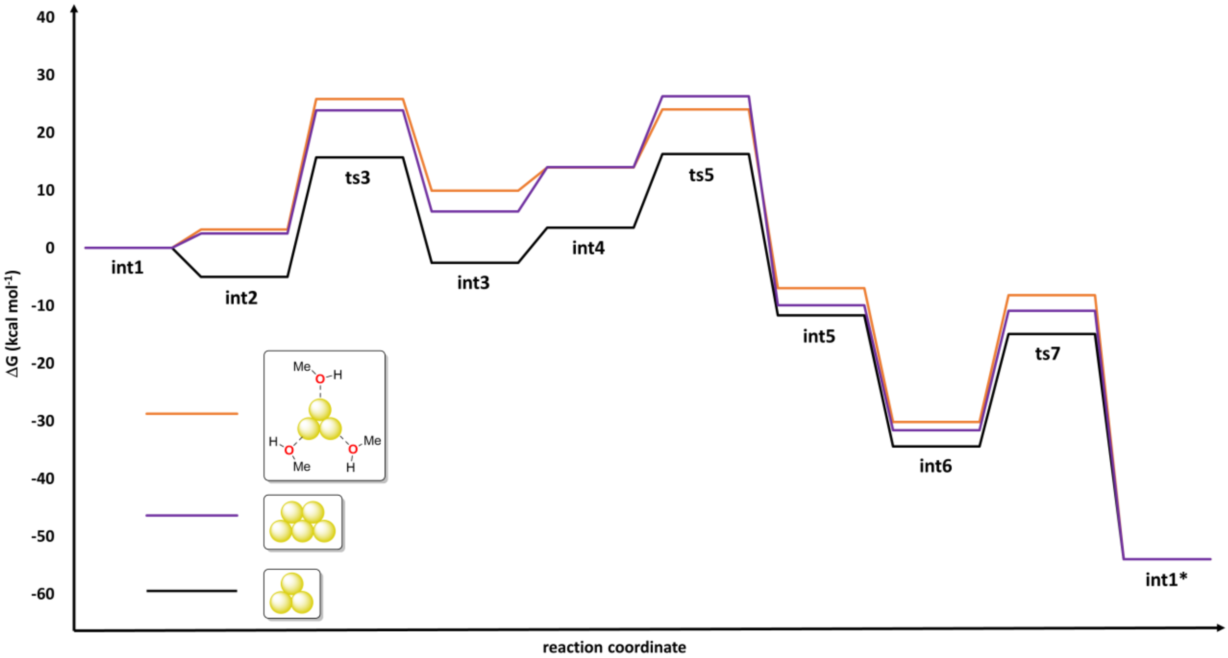

Supplementary Figure 7. Comparison between the complete minimum-energy reaction pathways calculated for different catalysts at M06-2X/SDD level. Gibbs free energies (ΔG) are in kcal mol^{-1}. The black pathway corresponds to the use of naked Au_3 as catalysts; the orange profile corresponds to the solvated (3 molecules of methanol) Au_3 catalyst and the purple profile corresponds to the use of naked Au_5 catalyst.

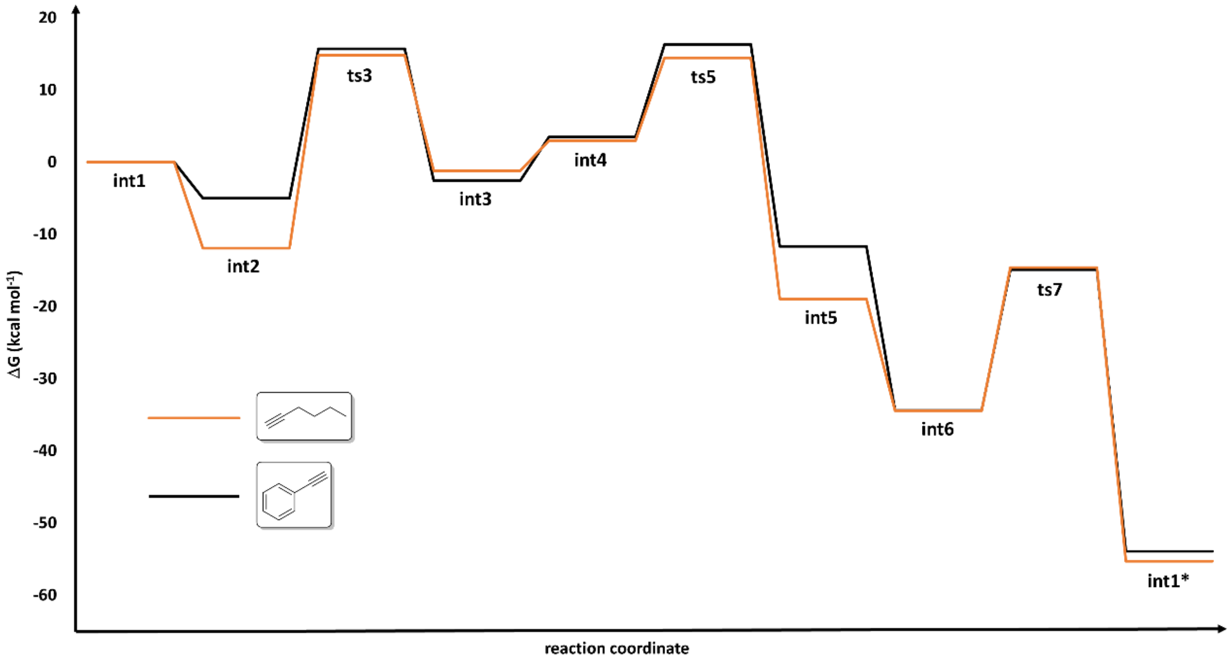

Supplementary Figure 8. Comparison between the complete minimum-energy reaction pathways for the Au_3-catalyzed hydration of phenylacetylene and 1-hexyne calculated with M06-2X/SDD. Relative Gibbs free energies (ΔG) are given in kcal mol^{-1}.

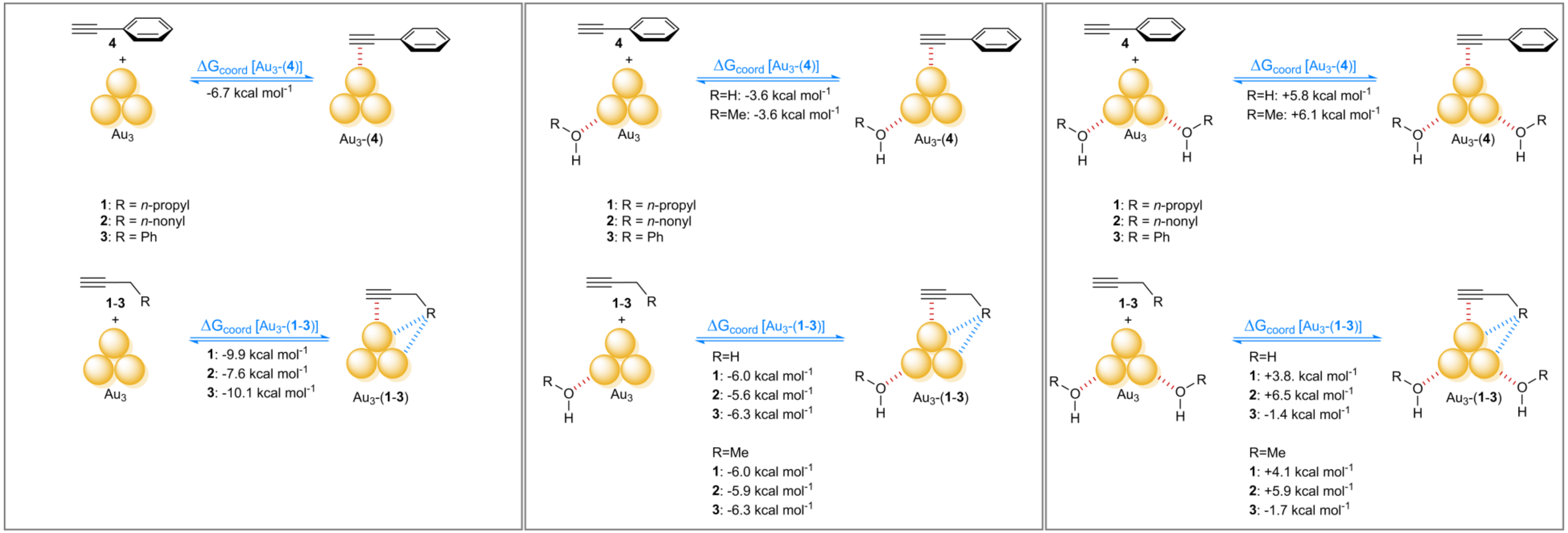

Supplementary Figure 9. Free energies of coordination (ΔG_{coord}) between unsolvated, monosolvated and disolvated (with either methanol or water) Au_3 subnanocluster and alkynes 1-4, calculated with M06-2X/SDD

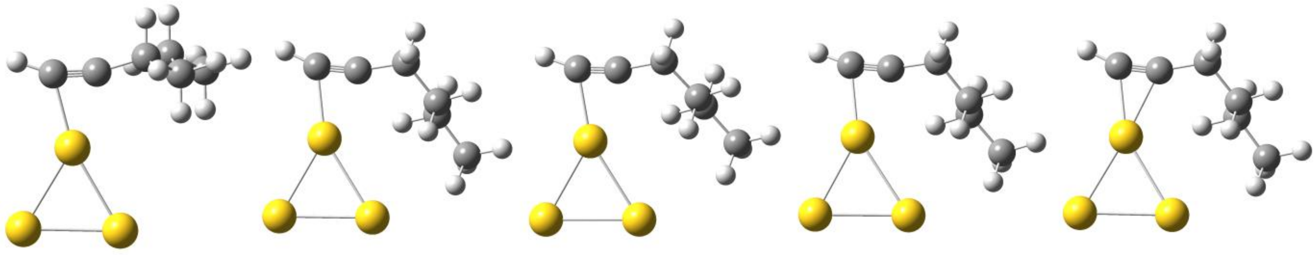

Supplementary Figure 10. Optimized model [Au_3-(1-hexyne)], in folded conformation, using different DFT functionals. With B3LYP the local minima showing the alkyl sidechain wrapping around the Au_3 cluster could not be located. In contrast, B3LYP-D3, ωB97xD, M06 and M06-2X did allow full optimization of such structures, which resulted to be more stable than the extended ones. In view of these results, M06-2X was selected for the whole study.

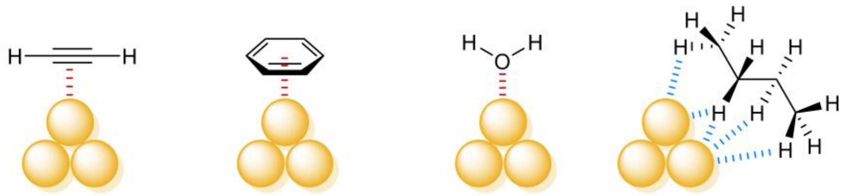

TOTAL	Au_3-acetylene	Au_3-benzene	Au_3-water	Au_3-butane
Electrostatic	-62.4 (25%)	-31.4 (24%)	-27.3 (31%)	-10.7 (19%)
Polarization	-78.2 (31%)	-32.2 (24%)	-19.1 (22%)	-8.5 (15%)
Dispersion	-24.1 (10%)	-23.6 (18%)	-13.1 (15%)	-19.5 (34%)
Exchange	-87.4 (35%)	-45.4 (34%)	-28.6 (32%)	-18.4 (32%)
Repulsion	+224.7	+115.7	+74.5	+48.5

Supplementary Figure 11: Energy Decomposition Analysis of different model Au3 adducts computed with M06-2X/SDD. The total interaction energy is divided into attractive (electrostatic, polarization, dispersion and exchange) and repulsive interactions. The percentage of each attractive interaction to the global attraction energy is shown in parentheses.

Supplementary Tables

Supplementary Table 1. Kinetics of phenylacetylene hydration catalyzed by Au subnanoclusters.

Alkyne	Pre-catalyst (% loading)	Time (hours)	Conversion (%)
Phenylacetylene (1)	AuCl (4% mol)	0.00	0
		0.25	13
		0.50	27
		0.75	35
		1.00	44
		1.50	52
		5.67	64
		8.67	68
		9.50	71
		14.50	78
		28.83	81
		53.00	89
		75.25	96
	AuCl (2% mol)	0.00	0
		1.17	8
		7.83	13
		26.33	24
		32.33	34
		50.58	41
		80.58	48
		105.08	60
		143.83	65
		168.08	77
		192.33	80
		216.50	87
		224.67	92
	$AuCl(PPh_3)$ (4% mol)	0.00	0
		1.67	9
		2.50	14
		25.00	59
		44.33	76
		65.75	96
	$AuCl(PPh_3)$ (2% mol)	0.00	0
		1.67	5

		2.50	8
		6.42	19
		57.17	37
		123.42	62
		160.75	77
		204.58	88
		234.58	90

Supplementary Table 2. Kinetics of 1-hexyne (1), 1-dodecyne (2) and benzylacetylene (3) hydration catalyzed by Au subnanoclusters.

Alkyne	Pre-catalyst (% loading)	Time (hours)	Conversion (%)
**1-hexyne (1)**	**AuCl (4% mol)**	0.00	0
		2.50	60
		4.67	87
		5.67	98
	**AuCl (2% mol)**	0.00	0
		1.00	27
		2.50	51
		5.00	54
		15.75	58
		72.00	73
**1-dodecyne (2)**	**AuCl (4% mol)**	0.00	0
		2.25	87
		3.50	94
		4.67	96
	**AuCl (2% mol)**	0.00	0
		1.00	42
		2.00	47
		5.83	54
		7.83	56
		23.00	58
		30.00	60
		48.42	62
		74.92	65
		97.00	68
		104.00	68
		144.00	72
		191.00	76

**Benzylacetylene (3)**	**AuCl (4% mol)**	0.00	0
		1.00	30
		2.17	56
		6.00	>99
	**AuCl (2% mol)**	0.00	0
		1.83	10
		6.50	21
		9.00	37
		23.08	79
		26.58	82
		30.42	95

Supplementary Table 3. Comparison of different DFT functionals.

Functional	G ([Au_3-(1-hexyne)]) –extended– (Hartree)[a]	G ([Au_3-(1-hexyne)]) –folded– (Hartree)[a]	ΔG (kcal mol^{-1})
**B3LYP**	-447.307727	-447.306952	**-0.49**
**B3LYP-D3**	-447.324973	-447.329090	**2.58**
**M06**	-447.162230	-447.166314	**2.56**
**M06–2X**	-446.749490	-446.753180	**2.32**
**ωB97xD**	-447.346628	-447.352445	**3.65**

[a] 1 Hartree = 627.51 kcal mol^{-1}

Supplementary Table 4. Electronic energies, entropies, enthalpies, Gibbs free energies and lowest frequencies of the lowest energy conformer of the calculated species.

**Compound**	**E_{elec} (Hartree)[a]**	**E_{elec} + ZPE[b] (Hartree)[a]**	**H (Hartree)[a]**	**S (cal mol^{-1} K^{-1})[c]**	**G (Hartree)[c]**	**Lowest freq. (cm^{-1})**
**1** –folded–	–39.931003	–39.788269	–39.779947	83.3	–39.819358	85.2
**1**–extended–	–39.930851	–39.788166	–39.779765	83.5	–39.819381	96.2
**2**–folded–	–81.139831	–80.825240	–80.809136	126.4	–80.866211	20.6
**2**–extended–	–81.143515	–80.829531	–80.812975	129.5	–80.870753	23.7
**3**	–55.745749	–55.606694	–55.598009	88.1	–55.638692	28.9
**4**	–48.879767	–48.768964	–48.761692	79.2	–48.799343	142.1
Au_3	–406.877060	–406.882168	–406.876116	90.265	–406.917957	42.2
Au_3-(**1**) –folded–	–446.694796	–446.708864	–446.693851	134.154	–446.753180	16.8
Au_3-(**1**) –extended–	–446.689986	–446.704420	–446.689042	140.187	–446.749490	13.1
Au_3-(**2**) –folded–	–487.727472	–487.749418	–487.726528	175.194	–487.800833	12.1

Au_3-(**2**) –extended–	–487.722398	–487.744758	–487.721454	184.080	–487.797560	5.8
Au_3-(**3**) –folded–	–462.513456	–462.528106	–462.512512	139.999	–462.572684	15.9
Au_3-(**3**) –extended–	–462.507035	–462.521820	–462.506091	148.094	–462.566757	4.9
Au_3-(**4**) –triangular–	–455.664181	–455.677340	–455.663237	132.865	–455.720979	11.7
Au_3-(**4**) –linear-TS–	–455.787965	–455.676211	–455.662700	134.1	–455.719431	–12.0
Au_3-(**4**) –linear–	–455.788342	–455.676588	–455.662088	140.2	–455.720682	6.0
Au_3-(**1**)·1H_2O	–464.109813	–463.942319	–463.996753	154.3	–463.990372	11.2
Au_3-(**1**)·2H_2O	–481.353764	–481.163665	–481.224982	177.3	–481.215007	11.6
Au_3-(**1**)·1MeOH	–470.962064	–470.764864	–470.821295	160.8	–470.814206	11.6
Au_3-(**1**)·2MeOH	–495.057652	–494.808217	–494.873893	191.3	–494.862350	13.1
Au_3-(**2**)·1H_2O	–505.325450	–504.985986	–505.048683	187.5	–505.041078	20.3
Au_3-(**2**)·2H_2O	–522.565611	–522.203530	–522.272558	209.1	–522.262074	10.4
Au_3-(**2**)·1MeOH	–512.177829	–511.809044	–511.874255	195.5	–511.865471	19.4
Au_3-(**2**)·2MeOH	–536.271168	–535.849962	–535.923969	224.6	–535.910833	16.4
Au_3-(**3**)·1H_2O	–479.925600	–479.761729	–479.817308	157.6	–479.810027	9.1
Au_3-(**3**)·2H_2O	–497.176925	–496.990247	–497.051564	177.9	–497.042487	9.5
Au_3-(**3**)·1MeOH	–486.777903	–486.584644	–486.643796	167.9	–486.634142	9.6
Au_3-(**3**)·2MeOH	–510.882724	–510.636457	–510.701562	190.6	–510.690872	17.4
Au_3-(**4**)·1H_2O	–473.055000	–472.919281	–472.972512	149.8	–472.966426	15.2
Au_3-(**4**)·2H_2O	–490.299561	–490.141071	–490.201368	172.7	–490.191710	10.9
Au_3-(**4**)·1MeOH	–479.907357	–479.742205	–479.799018	160.1	–479.790490	13.4
Au_3-(**4**)·2MeOH	–504.003403	–503.785789	–503.851083	188.4	–503.839100	15.2
Au_6	–813.876360	–813.888499	–813.875415	132.506	–813.933418	27.9
Au_6(**1**)$_2$	–893.477015	–893.508002	–893.476070	233.668	–893.569937	6.4
Au_6(**2**)$_2$	–975.560103	–975.606152	–975.559158	304.980	–975.680258	2.1
Au_6(**3**)$_2$	–925.120362	–925.152368	–925.119417	240.363	–925.214285	7.8
Au_6(**4**)$_2$	–911.434798	–911.464021	–911.433853	226.670	–911.523803	9.1

[a] 1 Hartree = 627.51 kcal mol^{-1}. [b] Zero-point energy obtained from vibrational frequencies. [c] Thermal corrections at 298.15 K.

Supplementary Methods

Definition of ΔG_{coord}, ΔG_{exch} and ΔG_{dim} (Figure 4) and how they were calculated.

ΔG_{coord} (coordination free energy) estimates the different coordination abilities of phenylacetylene and linear alkynes (1-hexyne, 1-docecyne) and benzylacetylene to Au_3 and Au_6 subnanoclusters. This energy has been calculated according to equation 1:

(1) $\Delta G_{coord} = G([Au_n\text{-alkyne}]) - \{G(Au_n) + G(\text{alkyne})\}$

ΔG_{exch} (exchange free energy) allows us to study the preference of each nanocluster (Au_3 or Au_6) to bind the different alkynes (typically with respect to phenylacetylene). This energy has been calculated according to equation 2:

(2) $\Delta G_{exch} = \{G([Au_n\text{-alkyne}]) + G(\text{alkyne'})\} - \{G([Au_n\text{-alkyne'}]) + G(\text{alkyne})\}$

ΔG_{dim} (dimerization free energy) takes into account the ability of each alkyne to stabilize small (Au_3) or bigger (Au_6) subnanoclusters. This energy has been calculated according to equation 3:

(3) $\Delta G_{dim} = G([Au_6\text{-(alkyne)}_2]) - 2\,G([Au_3\text{-alkyne}])$

Cartesian coordinates of the lowest energy calculated structures (all the optimized geometries can be obtained from the authors upon request)

Thermodynamic cycle

Au_3

Charge = 0 Multiplicity = 2

Au	0.000000	1.556000	0.000000
Au	-1.437200	-0.610300	0.000000
Au	1.437200	-0.945600	0.000000

Au_3-(1)-folded-

Charge = 0 Multiplicity = 2

C	1.779800	-2.832600	0.152700
C	2.520800	-1.939000	-0.272700
H	1.367800	-3.761500	0.519200
Au	-2.107100	-0.319600	0.186900
Au	-0.135500	1.616100	-0.270400
Au	0.409400	-1.134900	-0.033400
C	3.631600	-1.122300	-0.781000
H	4.552700	-1.555400	-0.360700
H	3.680500	-1.271100	-1.869300
C	3.558400	0.368000	-0.459700
H	4.445500	0.844400	-0.903900
H	2.679400	0.804900	-0.963700
C	3.496900	0.684000	1.025100
H	2.595400	0.217600	1.459800
H	4.359000	0.221500	1.533500
C	3.471400	2.178900	1.293600
H	2.591600	2.642000	0.817400
H	3.424900	2.395900	2.369600
H	4.368400	2.669400	0.887500

Au_3-(1)-extended-

Charge = 0 Multiplicity = 2

C	-2.032800	-2.329100	-0.000100
C	-2.527500	-1.196500	0.000100

H	-1.848100	-3.393100	-0.000200
Au	2.312000	-0.543500	-0.000400
Au	0.610900	1.702000	0.000200
Au	-0.299100	-0.961400	0.000100
C	-3.391500	-0.011800	0.000200
H	-3.146800	0.601000	0.881000
H	-3.146900	0.601200	-0.880500
C	-4.873400	-0.384000	0.000200
H	-5.090200	-1.005600	0.883100
H	-5.090200	-1.005400	-0.882800
C	-5.766300	0.846000	0.000300
H	-5.528100	1.464300	-0.880000
H	-5.528100	1.464200	0.880700
C	-7.243700	0.493000	0.000300
H	-7.506400	-0.101200	0.887800
H	-7.871900	1.393900	0.000400
H	-7.506500	-0.101100	-0.887400

Au_3-(2)-folded-

Charge = 0 Multiplicity = 2

C	-1.104300	3.377000	0.830100
C	0.080100	3.522000	0.499700
H	-2.110500	3.531600	1.192000
Au	-2.268600	-0.799700	0.189500
Au	0.227200	-0.899300	-1.057400
Au	-0.603600	1.399000	0.077400
C	1.439700	4.039000	0.288600
H	1.579000	4.254400	-0.781400
H	1.471300	5.005200	0.812800
C	2.551100	3.113700	0.792800
H	3.430000	3.727000	1.038300
H	2.225800	2.648100	1.737000
C	2.941500	2.039200	-0.211300
H	2.034100	1.623600	-0.684500
H	3.522800	2.495500	-1.030000
C	3.717200	0.889300	0.407400
H	3.115900	0.465500	1.227000
H	4.641200	1.265400	0.879300
C	4.065400	-0.194800	-0.605200
H	3.170300	-0.408700	-1.219600
H	4.812500	0.214400	-1.303400
C	4.574900	-1.508200	-0.006300
H	5.300600	-1.962100	-0.697300
H	5.134000	-1.298200	0.922300
C	3.486500	-2.542900	0.271600
H	2.944600	-2.740800	-0.671800
H	3.960000	-3.497900	0.553700
C	2.471300	-2.168700	1.340200
H	1.980100	-1.218000	1.072300
H	2.988500	-1.989900	2.299700
C	1.384800	-3.215200	1.528200
H	0.882300	-3.379100	0.557500
H	1.842600	-4.180600	1.800700
C	0.354100	-2.811200	2.568500
H	-0.134900	-1.865600	2.281400
H	-0.431600	-3.571500	2.683200
H	0.822900	-2.659500	3.552400

Au_3-(2)-extended-

Charge = 0 Multiplicity = 2

C	-1.033700	-1.453900	-0.339600
C	-1.169300	-0.226400	-0.393700
H	-1.170200	-2.525000	-0.317200
Au	3.609900	-1.075700	0.170900
Au	2.715100	1.583900	-0.077900
Au	1.015700	-0.655700	-0.146800
C	-1.639900	1.161100	-0.491900
H	-1.325000	1.699600	0.414900
H	-1.122900	1.641700	-1.334600
C	-3.156900	1.247100	-0.666300
H	-3.411900	2.306000	-0.823600
H	-3.445800	0.714200	-1.585700
C	-3.940500	0.702300	0.515900
H	-3.680500	-0.357600	0.677000
H	-3.634900	1.235000	1.432600
C	-5.445400	0.824400	0.336700
H	-5.736400	0.309000	-0.593100
H	-5.712400	1.886000	0.193300
C	-6.229000	0.261200	1.513200
H	-6.012700	-0.817100	1.606400
H	-5.857300	0.725900	2.440800
C	-7.735900	0.466300	1.424400
H	-8.202700	0.091600	2.349900
H	-7.955500	1.547400	1.391000
C	-8.397500	-0.212600	0.234100
H	-8.013100	0.215200	-0.706600
H	-8.115400	-1.280300	0.219500
C	-9.912900	-0.093200	0.245100

H	-10.193900	0.973900	0.277500
H	-10.307400	-0.536400	1.175900
C	-10.583300	-0.753200	-0.949400
H	-10.189100	-0.305500	-1.876500
H	-10.294600	-1.816800	-0.981800
C	-12.097500	-0.632500	-0.924100
H	-12.407900	0.423200	-0.920900
H	-12.559400	-1.115300	-1.796400
H	-12.514600	-1.101600	-0.020400

Au$_3$-(3)-folded-

Charge = 0   Multiplicity = 2

C	0.709900	3.333200	0.026500
C	1.637000	2.613800	-0.354500
H	0.077600	4.150200	0.341600
Au	-2.295700	-0.263900	0.238000
Au	0.161500	-1.485300	-0.361400
Au	-0.208500	1.322900	-0.050500
C	2.890900	1.999700	-0.805700
H	2.825400	1.832300	-1.889300
H	3.686500	2.745900	-0.649600
C	3.241100	0.704500	-0.105900
C	3.786300	-0.350400	-0.836900
C	3.034200	0.544600	1.265800
C	4.115600	-1.550100	-0.209900
H	3.946200	-0.237000	-1.913000
C	3.360400	-0.653500	1.893700
H	2.600500	1.363400	1.848500
C	3.900200	-1.705900	1.156600
H	4.536500	-2.370000	-0.796600
H	3.189100	-0.766700	2.966900
H	4.152800	-2.648000	1.648800

Au$_3$-(3)-extended-

Charge = 0   Multiplicity = 2

C	-1.963200	-1.924100	-0.161700
C	-2.297500	-0.739500	-0.044500
H	-1.931600	-2.998400	-0.269400
Au	2.577600	-0.783600	-0.024300
Au	1.223300	1.683000	0.076300
Au	-0.068200	-0.805300	-0.056900
C	-3.004000	0.542800	0.097000
H	-2.692900	1.005900	1.044900
H	-2.677400	1.217200	-0.706300
C	-4.504200	0.349600	0.059400
C	-5.167100	-0.212400	1.153100
C	-5.238400	0.714300	-1.068700
C	-6.544200	-0.404000	1.119100
H	-4.596800	-0.501000	2.041400
C	-6.618300	0.524600	-1.103000
H	-4.726000	1.155200	-1.928500
C	-7.273300	-0.035200	-0.010200
H	-7.053000	-0.841400	1.981400
H	-7.183700	0.818000	-1.990700
H	-8.355400	-0.183700	-0.036300

Au$_3$-(4) -triangular-

Charge = 0   Multiplicity = 2

C	-1.733700	-2.503400	-0.000000
C	-2.432000	-1.472500	-0.000000
H	-1.385700	-3.526700	-0.000000
C	-3.558900	-0.567100	-0.000000
C	-4.851100	-1.117200	0.000000
C	-3.393500	0.824300	0.000000
C	-5.960200	-0.279000	0.000000
H	-4.975200	-2.202600	0.000000
C	-4.510600	1.654200	0.000000
H	-2.383800	1.246600	-0.000000
C	-5.792600	1.106300	0.000000
H	-6.963700	-0.710800	0.000000
H	-4.376900	2.738500	0.000000
H	-6.667000	1.761600	0.000100
Au	2.368400	-0.602100	0.000000
Au	0.726200	1.660400	-0.000000
Au	-0.307900	-0.870700	-0.000000

Au$_3$-(4) -linear TS-

Charge = 0   Multiplicity = 2

C	-3.312984	-1.928337	0.234982
C	-3.367796	-0.699887	0.163629
H	-3.472582	-2.991657	0.333620
C	-3.677389	0.703853	0.073216
C	-4.937492	1.082199	-0.410986
C	-2.754906	1.682880	0.466106
C	-5.266543	2.428909	-0.497035
H	-5.651155	0.314607	-0.717263
C	-3.095677	3.026599	0.374836

H	-1.771836	1.383858	0.840109
C	-4.348520	3.401196	-0.105730
H	-6.248581	2.721427	-0.874741
H	-2.374980	3.787544	0.681743
H	-4.611282	4.459031	-0.176704
Au	1.433434	-1.026332	-0.096807
Au	2.355989	1.504546	0.018543
Au	-1.147673	-1.337192	0.054455

Au_3-(4) -linear-

Charge = 0 Multiplicity = 2

C	-3.472870	-1.952191	-0.490217
C	-3.746362	-0.765132	-0.326141
H	-3.433194	-3.013040	-0.686846
C	-4.245264	0.570712	-0.137838
C	-5.434132	0.759848	0.579838
C	-3.564484	1.672983	-0.672639
C	-5.934875	2.043845	0.754516
H	-5.957577	-0.103488	0.995649
C	-4.075016	2.951736	-0.490829
H	-2.635489	1.516301	-1.226913
C	-5.258187	3.138698	0.221136
H	-6.861318	2.190570	1.313762
H	-3.543739	3.809785	-0.907855
H	-5.655263	4.146362	0.362723
Au	1.157793	-0.627960	0.241587
Au	3.322780	0.936621	-0.081728
Au	-1.411285	-1.056376	-0.115270

Au_6

Charge = 0 Multiplicity = 1

Au	0.000000	0.000000	-1.673500
Au	0.000000	1.452100	0.838200
Au	0.000000	-1.452100	0.838200
Au	0.000000	0.000000	3.110500
Au	0.000000	2.692900	-1.556700
Au	0.000000	-2.692900	-1.556700

$Au_6(1)_2$

Charge = 0 Multiplicity = 1

Au	1.584400	0.837800	-0.276000
Au	-1.357800	1.064500	0.132800
Au	-0.026400	-1.430500	-0.035900
Au	0.331000	3.223400	-0.138700
Au	-2.775800	-1.171900	0.334800
Au	2.629100	-1.691700	-0.351100
C	-4.387400	-3.002600	0.965900
H	-3.924000	-3.528900	1.784700
C	-4.994500	-2.482300	0.046600
C	-5.785500	-1.886800	-1.030600
H	-5.102500	-1.590800	-1.842000
H	-6.436500	-2.673200	-1.439400
C	-6.624200	-0.687000	-0.586600
H	-7.353000	-1.014000	0.171600
H	-7.207500	-0.354100	-1.458600
C	-5.810000	0.477900	-0.046500
H	-5.269400	0.159700	0.861500
H	-5.034000	0.752800	-0.781800
C	-6.668600	1.689000	0.277600
H	-7.432800	1.439100	1.028400
H	-6.063000	2.514500	0.675800
H	-7.189700	2.054300	-0.619400
C	4.706300	-2.846700	-1.017000
C	5.116200	-2.343300	0.015200
H	4.453600	-3.336300	-1.943900
C	5.698800	-1.777400	1.233400
H	5.554700	-2.501400	2.048100
H	6.784600	-1.704100	1.066300
C	5.140900	-0.412500	1.638700
H	4.070300	-0.515100	1.884700
H	5.647200	-0.112800	2.568800
C	5.317600	0.671200	0.588000
H	4.791300	0.374400	-0.336200
H	6.384600	0.753300	0.323500
C	4.790900	2.016900	1.057400
H	4.897500	2.786200	0.280000
H	3.720900	1.949300	1.314800
H	5.327200	2.362600	1.953500

$Au_6(2)_2$

Charge = 0 Multiplicity = 1

Au	-1.438500	0.774200	0.654600
Au	1.466500	0.206200	0.936500
Au	-0.031600	-0.767800	-1.230800
Au	0.067600	1.701300	2.731700
Au	2.665500	-1.178300	-1.045500
Au	-2.738200	-0.344600	-1.419100
C	4.814500	-2.202000	-1.823800
H	4.948900	-2.967600	-1.076400

C	4.786600	-1.380400	-2.723100
C	4.801100	-0.445100	-3.850100
H	5.717400	0.161000	-3.781300
H	4.888700	-1.046100	-4.766600
C	3.573100	0.464200	-3.931700
H	2.666400	-0.160800	-3.865000
H	3.556100	0.929700	-4.927900
C	3.531200	1.541300	-2.856600
H	3.823700	1.106600	-1.884000
H	4.282800	2.317300	-3.076900
C	2.151400	2.160500	-2.709400
H	1.417300	1.342400	-2.589800
H	1.872600	2.686800	-3.638100
C	2.013300	3.095200	-1.519900
H	2.394300	2.580900	-0.617800
H	2.649700	3.986300	-1.658300
C	0.574000	3.514800	-1.270400
H	0.173500	3.996300	-2.178900
H	-0.036100	2.603900	-1.115800
C	0.403200	4.433100	-0.070900
H	0.921900	3.983300	0.793900
H	0.915100	5.390900	-0.265400
C	-1.050800	4.679100	0.317300
H	-1.482400	3.725800	0.672600
H	-1.080800	5.367000	1.179000
C	-1.934900	5.234700	-0.794800
H	-2.034700	4.484300	-1.596600
H	-2.950900	5.379100	-0.395500
C	-1.432100	6.544100	-1.382200
H	-1.292800	7.300800	-0.594600
H	-0.464700	6.415100	-1.890300
H	-2.139300	6.951800	-2.118000
C	-4.899600	-0.571200	-2.671300
C	-4.852700	-1.756800	-2.394900
H	-5.050800	0.452900	-2.973200
C	-4.844500	-3.196600	-2.127300
H	-4.941500	-3.705800	-3.097000
H	-5.748100	-3.443300	-1.549400
C	-3.597300	-3.693400	-1.393200
H	-2.704600	-3.319700	-1.923100
H	-3.567200	-4.790100	-1.469400
C	-3.536000	-3.272600	0.068500
H	-3.837400	-2.213900	0.160900
H	-4.273500	-3.846500	0.653600
C	-2.145300	-3.428200	0.661400
H	-1.422200	-2.950000	-0.024600
H	-1.867200	-4.495100	0.700300
C	-1.981700	-2.795200	2.032600
H	-2.355700	-1.755200	1.988900
H	-2.611200	-3.318200	2.773000
C	-0.535500	-2.772800	2.499300
H	-0.145100	-3.804800	2.521900
H	0.070000	-2.235500	1.744000
C	-0.341600	-2.107900	3.852700
H	-0.843700	-1.124600	3.837000
H	-0.857600	-2.698100	4.629000
C	1.119100	-1.894900	4.235200
H	1.559700	-1.161200	3.536300
H	1.163800	-1.428300	5.233700
C	1.980800	-3.153800	4.234400
H	2.070600	-3.539300	3.205400
H	3.002800	-2.879000	4.538500
C	1.460600	-4.253800	5.146500
H	1.330200	-3.883400	6.175200
H	0.485700	-4.634100	4.806800
H	2.153000	-5.106500	5.182100

$Au_6(3)_2$

Charge = 0 Multiplicity = 1

Au	1.497300	0.767500	-0.093900
Au	-1.497000	0.767900	0.093700
Au	-0.000100	-1.604700	0.000000
Au	0.000500	3.048700	-0.000100
Au	-2.711100	-1.663400	0.144600
Au	2.710700	-1.664400	-0.144600
C	-4.756400	-3.189700	0.492100
H	-4.328100	-4.046000	0.987900
C	-5.328000	-2.270900	-0.062900
C	-6.043500	-1.166000	-0.705300
H	-6.074000	-1.346100	-1.788300
H	-7.084500	-1.187000	-0.348900
C	-5.414900	0.181800	-0.418000
C	-4.869000	0.943600	-1.450100
C	-5.346500	0.661800	0.892400
C	-4.259800	2.167800	-1.178500
H	-4.911500	0.572400	-2.477800
C	-4.738600	1.882400	1.166200
H	-5.766800	0.066600	1.708700

C	-4.192600	2.639500	0.129900
H	-3.828500	2.752300	-1.994600
H	-4.688200	2.245800	2.195000
H	-3.711200	3.596600	0.343800
C	4.756800	-3.189300	-0.492700
C	5.327600	-2.270600	0.063400
H	4.329100	-4.045700	-0.988800
C	6.042300	-1.165800	0.706900
H	6.071400	-1.346000	1.789800
H	7.083700	-1.186700	0.351700
C	5.414000	0.182000	0.418900
C	4.868000	0.944200	1.450600
C	5.346200	0.661600	-0.891600
C	4.259000	2.168500	1.178500
H	4.909900	0.573300	2.478400
C	4.738700	1.882300	-1.166000
H	5.766700	0.066100	-1.707600
C	4.192400	2.639900	-0.130100
H	3.827600	2.753300	1.994200
H	4.688700	2.245500	-2.194900
H	3.711200	3.596900	-0.344400

Au_6 (4)$_2$

Charge = 0 Multiplicity = 1

Au	0.856100	1.487900	-0.373600
Au	-1.790000	0.292800	0.144100
Au	0.491200	-1.342000	-0.368000
Au	-1.445500	2.946400	0.126700
Au	-1.863700	-2.471800	0.065000
Au	2.943100	-0.064200	-0.841600
C	-4.065900	-3.838300	0.443000
H	-3.836900	-4.890000	0.382400
C	-4.437000	-2.681400	0.532800
C	-4.822800	-1.302200	0.627800
C	-4.735500	-0.632900	1.856800
C	-5.208000	-0.601700	-0.523800
C	-5.034600	0.722300	1.928100
H	-4.420200	-1.181100	2.747100
C	-5.504900	0.753300	-0.441000
H	-5.259900	-1.125700	-1.480400
C	-5.417500	1.416300	0.781700
H	-4.960700	1.242500	2.885300
H	-5.800300	1.297800	-1.340200
H	-5.645200	2.482900	0.840900
C	4.982900	-1.200300	-1.789800
C	5.381300	-0.929400	-0.669700
C	5.885800	-0.632400	0.642000
C	5.857600	0.683700	1.125900
C	6.405400	-1.660100	1.440200
C	6.347000	0.963000	2.395600
H	5.448600	1.479800	0.498600
C	6.892700	-1.368800	2.708500
H	6.422700	-2.683200	1.058800
C	6.864400	-0.060700	3.187200
H	6.324200	1.988600	2.770100
H	7.297400	-2.171200	3.329200
H	7.247800	0.163100	4.185200
H	4.731800	-1.471200	-2.802700

Reaction profile with Au_3 as a catalyst

int1

Charge = 0 Multiplicity = 2

Au	0.000000	1.556000	0.000000
Au	-1.437200	-0.610300	0.000000
Au	1.437200	-0.945600	0.000000

int2

Charge = 0 Multiplicity = 2

C	-1.733700	-2.503400	-0.000000
C	-2.432000	-1.472500	-0.000000
H	-1.385700	-3.526700	-0.000000
C	-3.558900	-0.567100	-0.000000
C	-4.851100	-1.117200	0.000000
C	-3.393500	0.824300	0.000000
C	-5.960200	-0.279000	0.000000
H	-4.975200	-2.202600	0.000000
C	-4.510600	1.654200	0.000000
H	-2.383800	1.246600	-0.000000
C	-5.792600	1.106300	0.000000
H	-6.963700	-0.710800	0.000000
H	-4.376900	2.738500	0.000000
H	-6.667000	1.761600	0.000100

Au	2.368400	-0.602100	0.000000
Au	0.726200	1.660400	-0.000000
Au	-0.307900	-0.870700	-0.000000

ts3

Charge = 0 Multiplicity = 2

C	-1.331500	-2.124300	-0.696700
H	-1.466600	-3.179800	-0.918600
C	-2.428600	0.233700	-0.292500
C	-2.347600	1.154300	-1.342700
C	-2.792000	0.658300	0.991500
C	-2.631500	2.495400	-1.107900
H	-2.061300	0.810400	-2.339400
C	-3.075400	2.000300	1.218300
H	-2.851800	-0.072700	1.801800
C	-2.997000	2.918500	0.169300
H	-2.568700	3.215300	-1.926700
H	-3.358000	2.334700	2.218700
H	-3.222700	3.971700	0.350600
C	-2.139800	-1.162400	-0.536300
Au	0.564400	-1.248600	-0.382700
Au	3.028600	-0.363500	-0.003600
Au	1.023800	1.574100	0.403300
O	-4.058100	-1.799400	-0.674600
H	-4.204700	-2.124800	0.261400
H	-4.638800	-0.990000	-0.783400
O	-5.494700	0.446500	-0.852300
C	-5.938300	0.948400	-2.100800
H	-5.099300	1.345600	-2.691400
H	-6.387900	0.107100	-2.640200
H	-6.697000	1.730700	-1.964200
H	-5.093200	1.166600	-0.324100
O	-4.327900	-2.418600	1.873900
H	-4.005700	-3.210700	2.343700
C	-5.245500	-1.697600	2.676900
H	-4.773100	-1.356400	3.608300
H	-5.555100	-0.820400	2.095600
H	-6.133700	-2.300200	2.909400

int3

Charge = 0 Multiplicity = 2

C	-1.672600	-1.740600	-0.504300
H	-1.974800	-2.795200	-0.555700
C	-2.602600	0.605200	-0.293500
C	-1.933400	1.321400	-1.290600
C	-3.197600	1.306500	0.762500
C	-1.855300	2.709600	-1.232400
H	-1.476000	0.774900	-2.119300
C	-3.111000	2.694000	0.825400
H	-3.723700	0.761400	1.552200
C	-2.442200	3.399400	-0.174000
H	-1.333500	3.256500	-2.021300
H	-3.569100	3.228300	1.661000
H	-2.380200	4.489100	-0.127300
C	-2.673200	-0.873100	-0.355600
O	-3.999600	-1.355500	-0.197100
Au	0.271000	-1.171800	-0.450000
Au	2.869000	-0.432500	-0.335900
Au	1.143100	1.434100	0.794700
H	-4.105300	-2.069000	0.602400
H	-4.794900	-0.669200	-0.407600
O	-5.791100	0.213400	-0.766800
C	-5.539200	1.178100	-1.787600
H	-4.970400	2.028700	-1.387600
H	-4.948700	0.669800	-2.557400
H	-6.489000	1.513300	-2.219800
H	-6.348700	0.581400	-0.051900
O	-4.101900	-2.922500	1.658500
H	-4.404800	-3.841400	1.514200
C	-3.037200	-2.852900	2.606600
H	-2.155000	-3.392400	2.239500
H	-2.795100	-1.791800	2.727100
H	-3.371200	-3.262900	3.566300

int4

Charge = -1 Multiplicity = 2

C	-1.881500	-2.269900	0.037500
H	-1.876000	-3.366900	-0.016500
C	-3.479200	-0.310000	0.114200
C	-4.539100	0.187200	-0.658000
C	-2.796100	0.578500	0.953400
C	-4.884900	1.534600	-0.614400
H	-5.085500	-0.486300	-1.324400
C	-3.142800	1.925300	0.998600
H	-1.984300	0.202400	1.580300
C	-4.186500	2.410400	0.213100
H	-5.704600	1.903100	-1.236100
H	-2.592900	2.600500	1.659700

H	-4.458300	3.468000	0.250500
C	-3.116500	-1.748200	0.036900
O	-4.207100	-2.593100	-0.099300
H	-5.005400	-2.167600	0.269500
Au	2.420100	-0.275300	0.003900
Au	0.477400	1.675800	-0.228900
Au	-0.123900	-1.274700	0.012900

ts5

Charge = -1 Multiplicity = 2

C	-2.199800	-0.917800	-0.808100
H	-2.755700	-1.852400	-0.975200
C	-2.373500	1.582600	-0.942300
C	-2.890600	2.531200	-0.055000
C	-1.292300	1.935400	-1.755000
C	-2.310400	3.791900	0.051900
H	-3.746900	2.261600	0.569400
C	-0.730400	3.207000	-1.672100
H	-0.892800	1.204000	-2.461700
C	-1.229300	4.133400	-0.759100
H	-2.707600	4.515400	0.768300
H	0.108300	3.473300	-2.320400
H	-0.778500	5.126100	-0.682800
C	-2.990900	0.214900	-0.981200
O	-4.281800	0.195000	-1.029500
Au	2.535700	-0.876500	-0.345000
Au	-0.162100	-0.923000	-0.629000
Au	1.207900	1.136400	1.057200
H	-4.792600	-0.681100	-0.079800
O	-5.022300	-1.274700	0.788900
C	-5.450700	-2.570400	0.432400
H	-6.406300	-2.521900	-0.108600
H	-4.710500	-3.085600	-0.201300
H	-5.599500	-3.159400	1.347000
H	-3.851700	-1.158200	1.460000
O	-2.818200	-0.920700	1.744800
C	-2.132600	-1.954400	2.427500
H	-2.204800	-2.907200	1.881200
H	-1.075500	-1.667000	2.509300
H	-2.555700	-2.072200	3.432500
H	-2.396500	-0.808900	0.627400

int5

Charge = -1 Multiplicity = 2

C	1.295900	2.216100	-1.405400
H	1.670600	1.831000	-2.356800
C	3.106800	0.891700	-0.180200
C	3.723600	0.628700	1.047200
C	3.350900	0.032000	-1.259100
C	4.552600	-0.479700	1.203000
H	3.527000	1.300200	1.885300
C	4.185000	-1.071800	-1.107000
H	2.877300	0.208200	-2.227200
C	4.782300	-1.335400	0.126500
H	5.019000	-0.679400	2.171100
H	4.365100	-1.735800	-1.956200
H	5.429800	-2.207800	0.246300
C	2.157600	2.057200	-0.264100
O	2.106300	2.829000	0.724300
H	0.869600	3.219900	-1.486000
Au	-2.110400	-0.997900	-0.305100
Au	-0.210600	0.811600	-0.828700
Au	0.435100	-1.700100	0.628700
H	0.669900	3.361000	1.075700
O	-0.268500	3.611700	1.356800
C	-0.857500	4.454700	0.393600
H	-0.170700	5.262700	0.093800
H	-1.162500	3.895100	-0.508200
H	-1.752000	4.915100	0.836100
H	-1.210900	2.393900	1.994600
O	-1.880100	1.724700	2.310900
C	-3.064400	1.939600	1.585000
H	-2.876800	1.987700	0.496600
H	-3.742200	1.092100	1.765700
H	-3.580700	2.865600	1.896000

int6

Charge = 0 Multiplicity = 2

C	-1.853600	1.478900	-1.849900
H	-1.730700	2.544000	-2.046600
C	-2.978800	-0.279400	-0.405300
C	-3.386600	-0.602900	0.892500
C	-2.993900	-1.269800	-1.395100
C	-3.776100	-1.902100	1.202200
H	-3.376200	0.168700	1.663500
C	-3.385400	-2.564200	-1.082900
H	-2.691100	-1.039300	-2.418200
C	-3.769200	-2.886800	0.218800

H	-4.082900	-2.146500	2.221600
H	-3.389700	-3.329800	-1.861700
H	-4.070600	-3.908100	0.462600
C	-2.489200	1.092500	-0.683400
O	-2.738600	1.949500	0.293900
H	-1.862300	0.818800	-2.714700
H	-2.200600	2.795200	0.187400
Au	0.335300	-1.837300	0.507500
Au	0.023300	0.754200	-0.872800
Au	2.410400	0.007700	-0.088600
H	-0.065400	2.763500	1.427200
O	0.310900	2.354500	2.234800
C	-0.572000	1.367100	2.714400
H	-0.739400	0.566100	1.972000
H	-0.111000	0.917600	3.603300
H	-1.548100	1.790600	3.003500
O	-1.086700	3.938800	0.279700
H	-0.585900	4.236800	-0.505200
C	-1.409500	5.047600	1.117100
H	-2.053400	5.759500	0.586400
H	-1.947800	4.641500	1.980600
H	-0.494700	5.545200	1.461800

ts7

Charge = 0 Multiplicity = 2

C	2.562800	-0.475100	-0.741900
H	2.602100	-1.514000	-0.386600
C	1.931300	1.906700	-0.046800
C	1.804500	2.827600	0.997600
C	1.528500	2.276200	-1.335200
C	1.270800	4.089400	0.764000
H	2.118300	2.530400	1.999600
C	1.005600	3.542900	-1.570400
H	1.611300	1.577900	-2.169900
C	0.868400	4.448700	-0.520700
H	1.165800	4.797400	1.589100
H	0.696000	3.821700	-2.580000
H	0.447600	5.439800	-0.705900
C	2.455100	0.542900	0.277900
O	2.856300	0.330700	1.439800
H	2.184700	-0.312800	-1.754000
Au	-1.630200	1.304000	0.333700
Au	0.142600	-0.910300	-0.369100
Au	-2.376300	-1.441200	-0.016400
H	4.086000	-0.636900	1.683500
O	4.973500	-1.114800	1.580000
C	4.896800	-2.455500	2.026300
H	5.869400	-2.927300	1.842700
H	4.685900	-2.489500	3.103500
H	4.120800	-3.019400	1.486300
H	5.249500	-0.804600	0.193800
O	5.177100	-0.488200	-0.808100
C	5.779200	-1.405200	-1.710000
H	6.848300	-1.490200	-1.479700
H	5.306800	-2.395800	-1.642400
H	5.660700	-1.011000	-2.725400
H	3.922000	-0.428500	-0.906200

Reaction profile with $Au_3(MeOH)_3$ as a catalyst

int1

Charge = 0 Multiplicity = 2

Au	1.399800	-0.213400	0.027000
Au	-0.975500	1.430300	-0.010100
Au	-1.347600	-1.100000	-0.010300
O	-1.672200	-3.378700	-0.057700
H	-2.387700	-3.755900	0.487000
C	-0.518600	-4.206800	-0.008700
H	-0.760800	-5.220700	-0.353100
H	0.217000	-3.760200	-0.687200
H	-0.099700	-4.242400	1.006400
O	-0.632000	3.705800	-0.058500
H	-1.248500	4.283800	0.427700
C	0.700200	4.185000	0.059600
H	0.772300	5.212000	-0.321700
H	1.045000	4.143700	1.101900
H	1.325600	3.526700	-0.553900
O	4.922500	-0.446200	-0.759200
H	5.777400	-0.436600	-1.231300
C	5.129900	-0.842800	0.579800
H	4.143300	-0.837400	1.063200
H	5.548700	-1.858500	0.646500
H	5.788900	-0.143900	1.117100

int2

Charge = 0 Multiplicity = 2

C	1.892000	-2.736500	0.013300
C	2.658600	-1.743300	0.008500
H	1.562900	-3.767000	0.022300
C	3.903300	-1.001300	0.012800
C	5.112200	-1.715800	0.049900
C	3.929900	0.399200	-0.019000
C	6.324400	-1.034600	0.054900
H	5.089800	-2.808000	0.074700
C	5.147700	1.073600	-0.014100
H	2.983700	0.948100	-0.047300
C	6.345100	0.360500	0.023000
H	7.260900	-1.596900	0.084000
H	5.160500	2.165900	-0.039200
H	7.299400	0.892900	0.027000
Au	-2.033700	-0.771100	-0.038200
Au	-0.303700	1.489900	-0.069700
Au	0.612000	-1.006900	-0.021800
O	-0.997400	3.806200	-0.083700
H	-1.687400	4.107700	-0.702700
C	-0.030600	4.826100	0.111200
H	0.488700	5.070500	-0.826700
H	-0.494800	5.731100	0.527600
H	0.697800	4.434500	0.831500
O	-4.444200	-0.733800	-0.064800
H	-4.948500	0.047700	0.225900
C	-5.182900	-1.919600	0.181500
H	-5.359400	-2.065400	1.256900
H	-6.141600	-1.902700	-0.355500
H	-4.575800	-2.751000	-0.196900

ts3

Charge = 0 Multiplicity = 2

C	-1.754000	-2.416700	-0.258100
H	-1.954100	-3.485500	-0.254300
C	-2.717800	0.024500	-0.340900
C	-2.533800	0.708200	-1.547300
C	-3.105000	0.722500	0.809800
C	-2.747800	2.081900	-1.603900
H	-2.226700	0.154500	-2.437800
C	-3.319200	2.094700	0.746100
H	-3.244700	0.176000	1.746000
C	-3.146800	2.774000	-0.461600
H	-2.606600	2.615100	-2.546500
H	-3.623100	2.639800	1.642500
H	-3.321500	3.851500	-0.509900
C	-2.526800	-1.411900	-0.288500
Au	0.223100	-1.612400	-0.166300
Au	2.712800	-0.835000	-0.053300
Au	0.824500	1.253700	0.132200
O	-4.439900	-1.938200	-0.274500
H	-4.624700	-2.011200	0.709000
H	-4.974300	-1.156600	-0.607000
O	-5.733700	0.249700	-1.073200
C	-6.079300	0.440500	-2.434300
H	-5.184800	0.608200	-3.052400
H	-6.577600	-0.476800	-2.767500
H	-6.772000	1.284900	-2.549700
H	-5.296000	1.052500	-0.722200
O	-4.807300	-1.898600	2.327700
H	-4.551400	-2.577400	2.980300
C	-5.720900	-0.975700	2.894700
H	-5.275200	-0.456900	3.754300
H	-5.950700	-0.237400	2.116600
H	-6.652400	-1.470800	3.199900
O	4.993600	-0.143000	0.087500
H	5.664500	-0.553800	-0.488500
C	5.215400	1.254500	0.184800
H	6.210700	1.463000	0.600500
H	5.107400	1.745100	-0.792900
H	4.452000	1.646700	0.867200
O	0.963600	4.950100	0.903100
H	1.452900	5.475800	0.240100
C	-0.359500	4.744300	0.457100
H	-0.929300	5.686400	0.418600
H	-0.388900	4.257900	-0.531100
H	-0.839700	4.068400	1.176700

int3

Charge = 0 Multiplicity = 2

C	-1.646400	-2.116900	0.709400
H	-1.862500	-2.911200	1.436600
C	-2.837800	-0.378800	-0.707900
C	-2.056200	-0.370800	-1.869400
C	-3.714300	0.691100	-0.480600
C	-2.133900	0.686900	-2.768700
H	-1.388300	-1.213200	-2.064800

C	-3.791100	1.749300	-1.381300
H	-4.341100	0.708700	0.416800
C	-2.999800	1.752200	-2.527300
H	-1.518800	0.674900	-3.671700
H	-4.472500	2.580200	-1.182000
H	-3.061900	2.582000	-3.235300
C	-2.735500	-1.490700	0.262900
O	-4.005900	-1.825500	0.838000
Au	0.242700	-1.431200	0.339200
Au	2.668200	-0.431100	-0.094200
Au	0.636500	1.477700	0.124000
H	-4.125300	-1.258400	1.738300
H	-4.815000	-1.776900	0.145800
O	-5.802700	-1.763700	-0.840800
C	-5.441100	-1.765500	-2.220500
H	-5.163400	-0.755700	-2.552300
H	-4.577700	-2.433000	-2.316100
H	-6.270900	-2.157400	-2.819900
H	-6.612200	-1.238800	-0.680600
O	-4.069300	-0.401400	2.821000
H	-4.501700	-0.696900	3.647100
C	-2.863800	0.317800	3.088900
H	-2.108600	-0.342700	3.533100
H	-2.497900	0.693700	2.125800
H	-3.079700	1.162200	3.753400
O	5.018300	0.132700	-0.530100
H	5.449900	0.988700	-0.361800
C	5.948800	-0.804700	-1.035800
H	6.754300	-0.999200	-0.313700
H	5.394800	-1.735500	-1.207300
H	6.378500	-0.467200	-1.989500
O	-1.013100	4.773400	1.110700
H	-0.612300	5.506300	0.603400
C	-1.980000	4.123000	0.314200
H	-2.853000	4.767500	0.122400
H	-1.560900	3.779000	-0.644600
H	-2.311200	3.235100	0.868800

int4

Charge = -1   Multiplicity = 2

C	2.241300	-2.266700	-0.224200
H	2.302000	-3.364600	-0.218700
C	3.725000	-0.227500	-0.223300
C	4.721900	0.305900	0.605400
C	2.996300	0.648900	-1.036100
C	4.953700	1.677700	0.650600
H	5.301900	-0.360100	1.251000
C	3.228300	2.019600	-0.992300
H	2.230500	0.244100	-1.701900
C	4.203600	2.541200	-0.144400
H	5.721400	2.075300	1.319200
H	2.636700	2.685200	-1.627400
H	4.383000	3.618600	-0.107900
C	3.449400	-1.685900	-0.214200
O	4.590600	-2.471600	-0.127500
H	5.360200	-1.977700	-0.470400
Au	-2.142400	-0.591000	0.166800
Au	-0.481700	1.486800	-0.560900
Au	0.414200	-1.372000	-0.109400
O	-4.548700	-0.041600	0.442000
H	-5.291100	-0.580600	0.766900
C	-5.006200	1.221500	-0.000800
H	-5.480600	1.784800	0.815300
H	-4.119900	1.771400	-0.342600
H	-5.708400	1.122700	-0.840800
O	0.501700	1.119700	3.014900
H	0.185300	1.822700	3.614900
C	1.636100	1.578000	2.308700
H	1.421000	2.496600	1.739300
H	2.490800	1.756300	2.980700
H	1.907300	0.791700	1.590700

ts5

Charge = -1   Multiplicity = 2

C	-2.124500	-1.417000	-0.711200
H	-2.312200	-2.496000	-0.832000
C	-3.222600	0.816400	-1.154500
C	-4.334900	1.517200	-0.672500
C	-2.134800	1.545000	-1.645500
C	-4.341900	2.907300	-0.637000
H	-5.192300	0.949700	-0.303100
C	-2.144900	2.938100	-1.623700
H	-1.272600	1.015700	-2.057100
C	-3.243400	3.623300	-1.110800
H	-5.209600	3.437700	-0.236600
H	-1.285300	3.490700	-2.012500
H	-3.246900	4.715900	-1.086200
C	-3.242300	-0.682400	-1.096600

O	-4.410900	-1.214900	-1.282700
Au	2.510400	-0.554000	-0.315900
Au	-0.165800	-0.830400	-0.445700
Au	1.077500	1.652600	0.502700
H	-4.655700	-2.122600	-0.310600
O	-4.756500	-2.716300	0.604100
C	-4.652500	-4.097100	0.339100
H	-5.512100	-4.437400	-0.255600
H	-3.726600	-4.339600	-0.208200
H	-4.651000	-4.646500	1.290300
H	-3.804300	-2.177900	1.359200
O	-2.940600	-1.605100	1.764100
C	-2.028800	-2.406200	2.492200
H	-1.724500	-3.290400	1.910000
H	-1.137300	-1.805600	2.716800
H	-2.494000	-2.728300	3.432200
H	-2.471400	-1.335600	0.694500
O	4.907100	-1.002200	-0.415800
H	5.456500	-0.875400	-1.209900
C	5.546700	-1.873000	0.498100
H	5.698200	-2.870800	0.062500
H	6.510600	-1.461700	0.828700
H	4.881900	-1.961600	1.365300
O	-1.484900	2.759700	2.379000
H	-0.658700	2.770500	1.839500
C	-2.004800	1.449400	2.334400
H	-2.194100	1.115800	1.301600
H	-1.330500	0.721600	2.817500
H	-2.958500	1.451800	2.878000

int5

Charge = -1   Multiplicity = 2

C	1.132000	2.850500	-1.161200
H	1.472100	2.671900	-2.182400
C	3.099500	1.492300	-0.288000
C	3.924400	1.184600	0.796500
C	3.267600	0.784400	-1.484100
C	4.889900	0.187100	0.696000
H	3.786900	1.735000	1.728700
C	4.231300	-0.212200	-1.586600
H	2.623600	0.988300	-2.342100
C	5.044700	-0.515700	-0.495900
H	5.522900	-0.046500	1.555700
H	4.344500	-0.761500	-2.524400
H	5.797700	-1.304100	-0.575900
C	2.034100	2.541500	-0.103000
O	1.957100	3.079700	1.033200
H	0.593700	3.791900	-1.028300
Au	-1.988500	-0.735900	-0.457800
Au	-0.231900	1.192900	-0.763300
Au	0.642600	-1.379800	0.321800
H	0.523900	3.360300	1.543800
O	-0.414700	3.466800	1.919500
C	-1.146300	4.385900	1.147400
H	-0.560300	5.296700	0.943800
H	-1.465700	3.949700	0.184600
H	-2.043400	4.679600	1.710100
H	-1.210000	2.103600	2.431000
O	-1.795300	1.339900	2.699900
C	-3.054300	1.539700	2.114600
H	-2.976300	1.780200	1.038800
H	-3.633400	0.608800	2.204100
H	-3.623200	2.344500	2.613600
O	-3.834600	-2.268200	-0.291900
H	-3.689000	-3.177300	0.027800
C	-5.100300	-1.792000	0.129700
H	-5.908400	-2.419100	-0.271200
H	-5.169100	-1.751900	1.226000
H	-5.203800	-0.776800	-0.271700
O	2.726000	-4.329500	1.518800
H	2.675500	-5.122500	0.950000
C	3.668000	-3.424600	0.982700
H	4.694200	-3.821800	1.038500
H	3.434900	-3.158900	-0.060700
H	3.609700	-2.506000	1.580800

int6

Charge = 0 Multiplicity = 2

C	-1.732100	2.111100	-1.245900
H	-1.383600	3.079100	-0.886100
C	-3.291600	0.163400	-0.896500
C	-4.437600	-0.222000	-0.194600
C	-2.923500	-0.547600	-2.045600
C	-5.215700	-1.283600	-0.647200
H	-4.720100	0.326600	0.705500
C	-3.693200	-1.617500	-2.484800
H	-2.012300	-0.280400	-2.587400
C	-4.844900	-1.985600	-1.790100

H	-6.115200	-1.568000	-0.096200
H	-3.388900	-2.171900	-3.375400
H	-5.450100	-2.825600	-2.139200
C	-2.512600	1.332500	-0.414000
O	-2.707900	1.605800	0.860000
H	-1.814100	1.974300	-2.322700
Au	0.122900	-1.574200	0.905400
Au	0.030500	0.786900	-0.733900
Au	2.341700	-0.346000	-0.397700
H	-2.053200	2.325300	1.218500
H	-0.827900	4.125600	1.665500
O	-1.000200	3.148800	1.805800
C	0.152100	2.506200	2.310100
H	0.385200	2.860000	3.324900
H	-0.052700	1.426900	2.347300
H	1.019500	2.668100	1.651700
O	4.520700	-1.215400	-0.257300
H	4.743300	-1.854400	0.443800
C	5.652700	-0.432700	-0.597500
H	6.479300	-1.072200	-0.934500
H	5.346400	0.220700	-1.422300
H	5.979100	0.186000	0.249800
O	-2.400200	-3.709700	2.282700
H	-2.447600	-4.587200	1.855500
C	-3.136500	-2.776200	1.521500
H	-2.802900	-2.738700	0.470800
H	-4.217300	-2.988400	1.549100
H	-2.960600	-1.786200	1.964000
O	-0.463500	5.666600	1.428400
H	0.129800	6.186800	2.002200
C	-0.916000	6.444900	0.334400
H	-1.456200	7.336200	0.680400
H	-0.080300	6.742900	-0.313000
H	-1.605500	5.816300	-0.240100

ts7

Charge = 0 Multiplicity = 2

C	3.018100	-0.470900	-0.717500
H	3.098900	-1.492300	-0.322800
C	2.185200	1.879100	-0.165800
C	2.096100	2.891000	0.795200
C	1.684400	2.116000	-1.451100
C	1.513300	4.113200	0.482300
H	2.484300	2.697800	1.796600
C	1.104700	3.340400	-1.764900
H	1.722100	1.339800	-2.217700
C	1.014800	4.339700	-0.798700
H	1.443700	4.893700	1.243600
H	0.711800	3.511500	-2.769500
H	0.550000	5.297400	-1.045300
C	2.773400	0.565000	0.245400
O	3.112200	0.431900	1.444100
H	2.680400	-0.373000	-1.750300
Au	-1.295300	1.065300	0.232300
Au	0.558600	-1.095300	-0.385700
Au	-1.912300	-1.735300	-0.081500
H	5.619000	-0.637400	0.426000
H	4.409900	-0.338200	-0.790600
O	5.626600	-0.350400	-0.598600
C	6.312200	-1.284100	-1.421300
H	6.278800	-0.918800	-2.453600
H	7.357300	-1.350500	-1.095900
H	5.844400	-2.277800	-1.366700
O	-4.135600	-2.304000	0.147400
H	-4.349300	-3.131300	0.617600
C	-5.036300	-1.271200	0.522900
H	-4.779300	-0.395400	-0.083700
H	-6.071000	-1.569600	0.309000
H	-4.927900	-1.018700	1.586700
O	-2.595200	4.505300	-0.245700
H	-3.033200	5.300500	-0.606000
C	-2.083300	4.791300	1.038100
H	-2.882700	5.054500	1.748000
H	-1.585900	3.876100	1.388100
H	-1.341000	5.604400	1.012500
O	5.239800	-0.907500	1.767300
C	5.141400	-2.240400	2.231000
H	6.122500	-2.715600	2.112400
H	4.868500	-2.255600	3.294800
H	4.397000	-2.815300	1.658700
H	4.328400	-0.442200	1.776200

Reaction profile with Au_5 as a catalyst

int1

Charge = 0 Multiplicity = 2

Au	-2.691400	0.946000	0.000000
Au	-1.394200	-1.466800	0.000000
Au	0.000000	1.041400	0.000000
Au	1.394200	-1.466700	0.000000
Au	2.691400	0.946100	0.000000

int2

Charge = 0 Multiplicity = 2

C	-4.739800	0.612500	-0.031300
C	-3.907900	1.506700	-0.020100
H	-5.564300	-0.083000	-0.044100
C	-2.903700	2.534800	-0.005900
C	-2.326000	2.965400	-1.210700
C	-2.448200	3.053900	1.215700
C	-1.304700	3.909200	-1.187900
H	-2.676400	2.545600	-2.156400
C	-1.426000	3.997300	1.226100
H	-2.894000	2.704200	2.149600
C	-0.853400	4.424400	0.027800
H	-0.852500	4.239400	-2.125700
H	-1.069800	4.398000	2.177800
H	-0.046200	5.160800	0.041400
Au	-0.413800	-2.241000	0.013300
Au	-0.035700	0.543600	-0.013800
Au	-2.551500	-0.672400	-0.005700
Au	2.270500	-1.219200	0.007500
Au	2.408400	1.601800	-0.002900

ts3

Charge = 0 Multiplicity = 2

C	-3.644400	-1.222500	-0.507400
H	-4.376800	-2.016700	-0.633800
C	-3.094500	1.336100	-0.353100
C	-2.930500	2.183400	-1.455200
C	-2.585200	1.710600	0.897500
C	-2.250900	3.387300	-1.307200
H	-3.335100	1.885900	-2.424800
C	-1.908300	2.917200	1.037600
H	-2.715600	1.041600	1.751600
C	-1.738500	3.754200	-0.063300
H	-2.116800	4.043400	-2.169900
H	-1.505100	3.202100	2.011800
H	-1.200700	4.698600	0.047900
C	-3.722900	0.041300	-0.505700
Au	-1.551200	-1.626800	-0.219700
Au	0.451100	0.384300	-0.080900
Au	1.009600	-2.322000	0.112700
Au	2.292600	2.305500	-0.071300
O	-5.588600	0.691000	-0.892300
H	-6.148200	-0.120800	-0.726800
H	-5.802000	1.351400	-0.169300
Au	3.226100	-0.386600	0.194800
O	-5.971200	2.436300	1.045300
H	-6.594200	2.367100	1.793000
C	-5.403100	3.732300	0.984300
H	-6.174700	4.498000	0.823800
H	-4.836300	3.959800	1.897800
H	-4.712500	3.735200	0.131700
O	-6.859400	-1.527400	-0.221600
H	-7.611400	-2.006700	-0.617500
C	-6.757800	-1.809800	1.163300
H	-5.892600	-1.247000	1.535900
H	-7.656100	-1.484800	1.706000
H	-6.585500	-2.880800	1.337100

int3

Charge = 0 Multiplicity = 2

C	-3.597400	-0.961300	-0.289100
H	-4.395200	-1.683200	-0.505200
C	-3.132800	1.521400	-0.048700
C	-3.169900	2.614200	-0.922800
C	-2.319900	1.590300	1.088800
C	-2.392600	3.742600	-0.675500
H	-3.809800	2.573800	-1.808100
C	-1.550900	2.722900	1.339400
H	-2.304300	0.747000	1.783700
C	-1.583200	3.800900	0.457000
H	-2.421600	4.583400	-1.372500
H	-0.922300	2.763300	2.232200
H	-0.976300	4.688000	0.653100
C	-3.956900	0.320400	-0.315700
O	-5.282200	0.714600	-0.658300
Au	-1.651100	-1.537100	-0.031000
Au	0.547700	0.334500	-0.092600
Au	0.927300	-2.358500	0.247500
Au	2.347700	2.276500	-0.321500
H	-5.927200	-0.072600	-0.991000
H	-5.709700	1.365600	0.076100

Au	3.322900	-0.427100	0.098800
O	-6.224100	2.197000	1.055400
H	-6.929500	1.828900	1.623700
C	-5.508100	3.239400	1.716000
H	-6.208100	4.018700	2.038200
H	-4.951100	2.842500	2.574000
H	-4.808200	3.655600	0.983800
O	-6.751500	-1.098300	-1.399300
H	-7.201900	-1.033700	-2.264200
C	-7.515200	-1.836600	-0.449900
H	-6.910800	-1.891400	0.461900
H	-8.464100	-1.330400	-0.234600
H	-7.696800	-2.850100	-0.825100

int4

Charge = -1   Multiplicity = 2

C	-4.187700	-0.283600	-0.349300
H	-5.062000	-0.847700	-0.700800
C	-3.407900	2.040800	0.228900
C	-3.254600	3.247500	-0.465700
C	-2.608100	1.815500	1.357400
C	-2.306100	4.185900	-0.065800
H	-3.868700	3.443900	-1.349400
C	-1.665000	2.755500	1.760700
H	-2.737500	0.890000	1.923300
C	-1.506200	3.942500	1.047200
H	-2.189800	5.113000	-0.632500
H	-1.049400	2.559200	2.642300
H	-0.761000	4.677200	1.361600
C	-4.400500	1.034100	-0.232000
O	-5.618300	1.576800	-0.608300
H	-5.743600	2.444500	-0.177600
Au	0.084400	-2.357500	0.137000
Au	0.070200	0.379800	-0.079800
Au	-2.364400	-1.163100	-0.119200
Au	2.108400	2.078400	-0.233200
Au	2.713800	-0.751900	0.068800

ts5

Charge = -1   Multiplicity = 2

C	-3.727400	-0.337700	-0.372400
H	-4.655400	-0.891300	-0.163600
C	-2.835900	1.787800	-1.379500
C	-2.725100	3.095900	-0.897800
C	-1.884600	1.323400	-2.293500
C	-1.663300	3.908300	-1.285600
H	-3.478200	3.467100	-0.197400
C	-0.835600	2.143500	-2.702900
H	-1.974600	0.310100	-2.692100
C	-0.716000	3.433600	-2.190800
H	-1.576200	4.920100	-0.881900
H	-0.103300	1.768700	-3.422400
H	0.115800	4.071400	-2.500000
C	-3.970100	0.930700	-0.893400
O	-5.121500	1.516000	-0.861200
Au	0.591000	-2.382100	-0.273800
Au	-1.899300	-1.284500	-0.329600
Au	0.380200	0.346400	0.155700
Au	2.256400	2.171200	0.555100
H	-5.742100	1.294700	0.358700
O	-6.013800	1.153200	1.392700
C	-7.019500	0.174600	1.536000
H	-7.952600	0.513600	1.064400
H	-6.722100	-0.785700	1.084000
H	-7.209400	0.014600	2.605600
H	-4.800000	0.882100	1.915900
O	-3.729100	0.665600	2.051800
C	-3.438300	-0.332700	3.012900
H	-4.007600	-1.253500	2.813300
H	-2.364500	-0.556600	2.960200
H	-3.682100	0.041900	4.014500
H	-3.540500	0.246100	0.946000
Au	3.095700	-0.591300	0.098000

int5

Charge = -1   Multiplicity = 2

C	-4.145500	0.276700	-1.158300
H	-3.990700	0.553400	-2.202200
C	-2.874700	2.341400	-0.389100
C	-2.616200	3.246900	0.642900
C	-2.140000	2.452900	-1.578400
C	-1.645500	4.234800	0.498900
H	-3.185700	3.165300	1.570900
C	-1.175700	3.443300	-1.726200
H	-2.302400	1.746900	-2.395200
C	-0.921800	4.335500	-0.685300
H	-1.451800	4.929200	1.319900
H	-0.607400	3.509300	-2.656800

H	-0.155500	5.106000	-0.798600
C	-3.892100	1.252300	-0.153800
O	-4.441200	1.210000	0.979700
H	-5.025800	-0.341900	-0.970500
Au	-0.119700	-2.281900	-0.176500
Au	-2.334200	-0.859600	-0.657400
Au	0.140100	0.498000	-0.106800
Au	2.567100	-1.120700	0.063800
Au	2.542800	1.710800	-0.130400
H	-3.448700	0.794700	2.137200
O	-2.797200	0.415400	2.806200
C	-3.090500	-0.946900	3.011300
H	-4.123100	-1.083700	3.368000
H	-2.948000	-1.538000	2.089500
H	-2.407700	-1.333900	3.779700
H	-1.230600	0.899100	2.681900
O	-0.259900	1.137200	2.654100
C	0.480300	0.083300	3.213300
H	0.258300	-0.882900	2.724000
H	1.548500	0.294500	3.057200
H	0.301300	-0.018800	4.297700

int6

Charge = 0 Multiplicity = 2

C	-4.185500	0.947900	-0.748100
H	-4.487300	1.958100	-0.471100
C	-3.913200	-1.470600	-0.122900
C	-3.324700	-2.290800	0.849700
C	-4.169300	-1.996100	-1.392200
C	-2.972900	-3.604600	0.545000
H	-3.139800	-1.884900	1.845600
C	-3.824900	-3.310300	-1.690400
H	-4.648800	-1.379800	-2.155400
C	-3.218300	-4.115000	-0.728000
H	-2.507800	-4.231100	1.309500
H	-4.034900	-3.710600	-2.684600
H	-2.943900	-5.144600	-0.968000
C	-4.155200	-0.042200	0.204000
O	-4.286000	0.195800	1.498200
H	-4.190000	0.688200	-1.805000
Au	-0.493300	-1.517000	0.226900
Au	-1.836400	0.898900	-0.486500
Au	0.800200	0.941100	-0.336600
Au	2.288300	-1.432800	0.371400
Au	3.577200	0.894700	-0.140300
H	-3.975000	4.805900	-0.160800
O	-3.232400	4.277700	0.189200
C	-2.125500	4.320300	-0.695000
H	-2.350200	3.800000	-1.637900
H	-1.828200	5.357600	-0.900200
H	-1.295800	3.803100	-0.196400
H	-4.060100	1.184000	1.727800
O	-3.521300	2.503100	2.077700
H	-3.437000	3.182000	1.344300
C	-2.324500	2.464700	2.833600
H	-1.469800	2.148700	2.212800
H	-2.116100	3.449000	3.275200
H	-2.461300	1.737700	3.643300

ts7

Charge = 0 Multiplicity = 2

C	4.191800	-0.959300	-0.711200
H	4.194400	-1.979900	-0.311300
C	3.475300	1.410900	-0.185100
C	2.825500	2.196400	0.777600
C	3.525200	1.853200	-1.507700
C	2.196400	3.386400	0.408000
H	2.816600	1.857500	1.815000
C	2.923200	3.056800	-1.869800
H	4.042100	1.261700	-2.266700
C	2.246600	3.816700	-0.920900
H	1.686900	3.988600	1.162800
H	2.977800	3.398300	-2.905500
H	1.760000	4.750500	-1.209900
C	4.015700	0.082300	0.246000
O	4.319300	-0.050700	1.456600
H	3.800200	-0.841100	-1.724500
Au	0.267000	1.271600	0.241800
Au	1.485600	-1.198000	-0.479200
Au	-1.143400	-1.126900	-0.259300
Au	-2.511100	1.327800	0.426000
Au	-3.874100	-0.990400	-0.033600
H	5.615100	-0.947800	-0.808400
H	6.773600	-1.240800	0.437100
O	6.804400	-0.990700	-0.607300
C	7.393400	0.286400	-0.818100
H	8.340100	0.342000	-0.268000
H	7.585300	0.407300	-1.889600

H	6.721100	1.086300	-0.471100
O	6.423600	-1.428300	1.772500
C	6.371100	-2.728400	2.328700
H	7.360500	-3.186000	2.212800
H	6.130100	-2.673700	3.398500
H	5.625400	-3.356300	1.818700
H	5.514100	-0.960600	1.796200

Reaction profile of 1-hexyne hydration

int1

Charge = 0 Multiplicity = 2

Au	0.000000	1.555957	0.000000
Au	1.437234	-0.945631	0.000000
Au	-1.437234	-0.610325	0.000000

int2

Charge = 0 Multiplicity = 2

C	1.779797	-2.832573	0.152673
C	2.520845	-1.938950	-0.272715
H	1.367796	-3.761502	0.519219
Au	-2.107103	-0.319575	0.186894
Au	-0.135483	1.616090	-0.270424
Au	0.409442	-1.134863	-0.033396
C	3.631551	-1.122322	-0.781018
H	4.552687	-1.555416	-0.360700
H	3.680474	-1.271143	-1.869334
C	3.558405	0.367962	-0.459721
H	4.445454	0.844396	-0.903886
H	2.679421	0.804897	-0.963745
C	3.496943	0.683976	1.025122
H	2.595357	0.217623	1.459806
H	4.359025	0.221515	1.533549
C	3.471350	2.178890	1.293604
H	2.591578	2.641974	0.817430
H	3.424853	2.395854	2.369562
H	4.368376	2.669381	0.887548

ts3

Charge = 0 Multiplicity = 2

C	-2.091279	-0.980925	-0.572676
C	-2.551638	0.127724	-0.173242
H	-2.552078	-1.872408	-0.992914
Au	2.641234	-1.009962	-0.197960
Au	1.559981	1.431192	0.689009
Au	-0.000419	-0.835898	-0.344200
C	-2.410194	1.492736	0.353499
H	-3.355974	1.800947	0.818348
H	-1.655783	1.443808	1.155105
C	-1.970906	2.502912	-0.705317
H	-1.795867	3.465754	-0.198769
H	-0.994231	2.182624	-1.108984
C	-2.965753	2.687270	-1.838861
H	-3.145062	1.713941	-2.323516
H	-3.937168	2.998249	-1.421298
C	-2.490362	3.700309	-2.866166
H	-1.535979	3.387010	-3.315221
H	-3.219110	3.824030	-3.678915
H	-2.330822	4.686305	-2.404733
O	-4.591869	0.146108	-0.430936
H	-4.958986	0.265552	0.489372
H	-4.796535	-0.790141	-0.708630
O	-4.939790	-2.436464	-0.983300
H	-5.486810	-2.844316	-1.681179
O	-5.196186	0.459728	2.120593
H	-5.946088	0.854679	2.603500
C	-4.911016	-3.260809	0.169475
H	-4.510703	-4.256068	-0.066335
H	-4.241986	-2.773125	0.889567
H	-5.908939	-3.357749	0.619107
C	-4.235315	-0.057008	3.024220
H	-3.824383	0.735297	3.665102
H	-4.660973	-0.856075	3.646479
H	-3.423849	-0.477748	2.416414

int3

Charge = 0 Multiplicity = 2

C	-1.738577	-1.520051	-0.448580
H	-2.078603	-2.453170	-0.916758
C	-2.692849	-0.648509	-0.131449
O	-4.052664	-0.985751	-0.405474
H	-4.283492	-2.012614	-0.202866
H	-4.794722	-0.283795	-0.110532
Au	0.232036	-1.107409	-0.221954
Au	2.890013	-0.636999	0.048232
Au	1.330601	1.659401	0.090016

O	-5.749703	0.681356	0.187965
O	-4.511326	-3.306851	0.182551
C	-6.644489	1.174144	-0.803682
H	-6.897269	2.219832	-0.592217
H	-6.114540	1.115691	-1.760199
H	-7.552950	0.561339	-0.847657
H	-6.130513	0.734838	1.086253
H	-4.655712	-4.002174	-0.489648
C	-3.824434	-3.801398	1.329881
H	-2.830736	-4.177896	1.056345
H	-3.720074	-2.955222	2.017435
H	-4.418283	-4.588777	1.807656
C	-2.568159	0.726586	0.443240
H	-1.598671	0.788580	0.958228
H	-3.350710	0.881624	1.202578
C	-2.630944	1.825144	-0.617862
H	-1.814365	1.643040	-1.336264
H	-3.574550	1.749364	-1.185591
C	-2.497247	3.229510	-0.043252
H	-1.553523	3.289647	0.527375
H	-2.391986	3.940939	-0.876764
C	-3.664706	3.655597	0.833482
H	-4.612912	3.600751	0.276554
H	-3.765564	3.017708	1.723793
H	-3.542279	4.689732	1.184037

int4

Charge = -1   Multiplicity = 2

C	1.913205	-2.363843	0.184116
H	1.990455	-3.357248	0.645015
C	3.079050	-1.817896	-0.182643
O	4.259826	-2.502164	0.054083
H	5.005897	-2.042628	-0.374603
Au	-2.308334	-0.183025	-0.021110
Au	-0.208829	1.614098	-0.040174
Au	0.147235	-1.387066	0.079723
C	3.264192	-0.454601	-0.789796
H	2.369863	-0.206033	-1.379352
H	4.113784	-0.470911	-1.495565
C	3.481262	0.647344	0.244079
H	2.607166	0.658655	0.918563
H	4.359533	0.417406	0.871251
C	3.642283	2.021262	-0.386172
H	2.802457	2.189020	-1.083112
H	4.558786	2.040369	-0.998472
C	3.672199	3.139311	0.641398
H	3.817796	4.121702	0.170970
H	2.723141	3.171210	1.200703
H	4.483704	2.989757	1.369336

ts5

Charge = -1   Multiplicity = 2

C	-2.464742	-0.047932	-0.362405
H	-3.092493	-0.541957	-1.120351
C	-2.930872	1.226349	-0.041119
O	-4.123540	1.640697	-0.327874
H	-4.997198	0.652088	-0.249371
O	-5.629268	-0.225639	0.013878
H	-4.832050	-0.878278	0.780443
O	-3.952741	-1.289224	1.383079
H	-3.126473	-0.712368	0.764026
C	-3.772768	-2.684026	1.224768
H	-3.590959	-2.941925	0.169045
H	-2.910763	-3.003397	1.824221
H	-4.668939	-3.210792	1.576742
C	-6.095339	-0.911291	-1.127168
H	-6.792996	-0.276281	-1.690754
H	-5.267963	-1.204989	-1.794135
H	-6.630008	-1.817339	-0.811449
Au	2.014393	-1.657207	-0.062495
Au	-0.512027	-0.616158	-0.213279
Au	1.942056	1.097727	0.121931
C	-2.076697	2.201794	0.729667
H	-2.726398	2.810971	1.375948
H	-1.369732	1.646777	1.365604
C	-1.279598	3.115359	-0.197889
H	-1.967442	3.665082	-0.862320
H	-0.642711	2.489437	-0.847637
C	-0.400510	4.099419	0.557108
H	-1.033679	4.764012	1.167381
H	0.230775	3.536431	1.266592
C	0.486694	4.918764	-0.364082
H	-0.111862	5.479573	-1.097622
H	1.098214	5.640803	0.194963
H	1.170785	4.260291	-0.923646

int5

Charge = -1   Multiplicity = 2

C	-1.530919	-2.110898	-1.588990
H	-1.696384	-1.708412	-2.594051
C	-2.579066	-1.813465	-0.651387
O	-2.819404	-2.522112	0.352284
H	-1.573122	-3.214736	0.982447
O	-0.744095	-3.576760	1.440671
H	0.252246	-2.455492	2.157989
O	0.954091	-1.864173	2.551295
H	-1.203897	-3.155155	-1.581977
C	2.197148	-2.316888	2.083817
H	2.211388	-2.421566	0.983721
H	2.962787	-1.575006	2.353876
H	2.483784	-3.284374	2.533179
C	-0.132613	-4.555284	0.636529
H	-0.872743	-5.266971	0.237024
H	0.417815	-4.106909	-0.208847
H	0.580759	-5.118627	1.253785
Au	2.183665	0.567353	-0.131922
Au	0.011837	-0.869571	-0.803180
Au	-0.218110	1.909244	0.243763
C	-3.405644	-0.566293	-0.852180
H	-2.958520	0.044341	-1.650890
H	-4.402548	-0.880665	-1.205213
C	-3.540821	0.254306	0.423626
H	-2.541561	0.358932	0.884695
H	-4.161073	-0.289116	1.152595
C	-4.111794	1.640762	0.179091
H	-3.480713	2.156859	-0.565521
H	-5.113477	1.554183	-0.273349
C	-4.176661	2.475127	1.446654
H	-3.167282	2.610130	1.868184
H	-4.598461	3.472088	1.257768
H	-4.795403	1.985561	2.213445

int6

Charge = 0 Multiplicity = 2

C	2.420774	-0.234813	-1.916312
H	2.755064	-1.253468	-2.118875
C	2.897332	0.426214	-0.798802
O	3.536179	-0.190603	0.179017
H	2.109720	0.367793	-2.769596
Au	-1.419265	1.461976	0.248130
Au	0.496544	-0.463157	-0.840730
Au	-1.849031	-1.307694	0.007424
H	3.408391	-1.190604	0.122602
H	1.422976	-2.099875	1.462448
O	1.060820	-1.996857	2.366983
C	1.111117	-0.638534	2.740246
H	0.365691	-0.034498	2.192337
H	0.882185	-0.582990	3.812211
H	2.109460	-0.202994	2.572479
O	2.874153	-2.692583	0.236969
H	2.508662	-3.174955	-0.530763
C	3.625271	-3.572034	1.070528
H	4.488081	-3.978064	0.528150
H	3.973239	-2.975110	1.920421
H	2.989244	-4.386614	1.438623
C	2.837641	1.911091	-0.634378
H	3.855333	2.288115	-0.837235
H	2.181163	2.318837	-1.414411
C	2.403773	2.360618	0.755613
H	3.105485	1.949516	1.495216
H	1.414722	1.921341	0.980140
C	2.327471	3.875079	0.904121
H	2.134662	4.107551	1.962327
H	3.312194	4.313189	0.671511
C	1.255286	4.530452	0.046715
H	1.190618	5.608768	0.246984
H	1.454093	4.409288	-1.028313
H	0.266252	4.087787	0.252480

ts7

Charge = 0 Multiplicity = 2

C	2.610531	0.065428	-0.533046
H	2.752911	-1.019851	-0.637323
C	2.537648	0.591774	0.810362
O	3.048441	-0.012746	1.773627
H	2.137402	0.597980	-1.363015
Au	-1.813453	1.244123	0.199281
Au	0.277683	-0.726384	-0.298428
Au	-2.165655	-1.582070	-0.062967
H	4.354922	-0.872709	1.536232
H	5.385131	-0.355797	0.009885
O	5.255330	-1.189717	1.194350
C	5.315960	-2.600983	1.121121
H	6.283059	-2.874295	0.682544
H	5.247317	-3.041096	2.125141
H	4.512168	-3.009881	0.489999

O	5.204478	0.306483	-0.785330	C	1.484535	2.790872	-0.091017
C	5.736796	-0.171606	-2.011752	H	2.359361	3.039888	-0.714428
H	5.295352	-1.141425	-2.284665	H	0.787530	2.227850	-0.735128
H	5.510513	0.564816	-2.790858	C	0.789679	4.067729	0.356495
H	6.825063	-0.275875	-1.920967	H	1.492231	4.683891	0.940509
H	3.929321	0.298042	-0.750518	C	0.228938	4.865544	-0.807437
C	1.904074	1.932027	1.088061	H	-0.247683	5.795936	-0.469680
H	2.594132	2.477530	1.750094	H	1.019497	5.131757	-1.524709
H	1.018095	1.704802	1.709379	H	-0.528351	4.275789	-1.348368
